# Quantifying cell-generated forces: Poisson’s ratio matters

**DOI:** 10.1038/s42005-021-00740-y

**Published:** 2021-11-04

**Authors:** Yousef Javanmardi, Huw Colin-York, Nicolas Szita, Marco Fritzsche, Emad Moeendarbary

**Affiliations:** 1 Department of Mechanical Engineering, University College London, London WC1E 7JE, UK; 2 Kennedy Institute for Rheumatology, University of Oxford, Roosevelt Drive, Oxford OX3 7LF, UK; 3 Department of Biochemical Engineering, University College London, London WC1E 6BT, UK; 4 Rosalind Franklin Institute, Harwell Campus, Didcot OX11 0FA, UK; 5 Department of Biological Engineering, Massachusetts Institute of Technology, Cambridge, MA, USA

## Abstract

Quantifying mechanical forces generated by cellular systems has led to key insights into a broad range of biological phenomena from cell adhesion to immune cell activation. Traction force microscopy (TFM), the most widely employed force measurement methodology, fundamentally relies on knowledge of the force-displacement relationship and mechanical properties of the substrate. Together with the elastic modulus, the Poisson’s ratio is a basic material property that to date has largely been overlooked in TFM. Here, we evaluate the sensitivity of TFM to Poisson’s ratio by employing a series of computer simulations and experimental data analysis. We demonstrate how applying the correct Poisson’s ratio is important for accurate force reconstruction and develop a framework for the determination of error levels resulting from the misestimation of the Poisson’s ratio. In addition, we provide experimental estimation of the Poisson’s ratios of elastic substrates commonly applied in TFM. Our work thus highlights the role of Poisson’s ratio underpinning cellular force quantification studied across many biological systems.

Cell mechanics and mechanobiology are primarily built upon an understanding of the material properties of living cells, as well as their force mechanosensation^1–7^, generation, and transmission abilities^8–11^. Accordingly, there has been a growing demand for methodologies with sufficient sensitivity to robustly quantify cell generated mechanical forces^12–14^. Traction force microscopy (TFM) is one of the most powerful and widely used force quantification modalities applied to study a broad range of cellular phenomena^14,15^. TFM has significantly benefited from recent progress in super-resolution optical imaging modalities and computational techniques^16–18^. Nevertheless, as the sensitivity of TFM continues to improve, there is a pressing need to advance our understanding of the force–displacement relationship, which relies on the mechanical properties of the substrates used in TFM. A typical TFM experiment involves a cell that is adhered to an elastic substrate containing fluorescent beads (Fig. 1a). Fluorescent beads act as fiducial markers, allowing the displacements generated under the influence of cellular forces to be tracked by fluorescent time-lapse imaging. Typically, mechanically tuneable materials such as hydrogels or silicone elastomers are employed as substrates or embedding matrices that mimic the mechanical environment of the cells and report on the cell generated forces.

Owing to their biocompatibility and linear isotropic elastic behaviour, polymer-based gels are well suited to investigate the mechanical interactions between cells and their environment^11,19^. They primarily behave in an elastic manner under cell induced deformations, independent of the directionality and spatial position of the applied stress^20,21^. Isotropic linear elastic materials are fully characterised by two fundamental material parameters: The Young’s modulus (E) and the Poisson’s ratio (*ν*). The Young’s modulus defines the elastic behaviour of a material or a cell in the loading direction, while the Poisson’s ratio describes the degree to which the material or cell contracts or expands in the transverse direction, perpendicular to the loading direction^22,23^. While incompressible materials (ones that maintain their volume under load, i.e., *ν* = 0.5) are rare in nature, most hydrogels and silicone elastomers have Poisson’s ratio values ranging between 0.25 and 0.49, depending on their specific composition and method of preparation^24–28^.

While the spatiotemporal resolution of displacement measurements has been substantially increased thanks to the recent advancements in super-resolution microscopy technologies^16,29^, there is an emerging need to improve our understanding of the mechanical properties of the gels used for TFM. Previous works have primarily focused on the quantification of the Young’s modulus, using methods such as atomic force microscopy (AFM), to investigate its effects on cellular responses and estimate the cellular forces^30^. However, the role of the Poisson’s ratio and its impact on mechanical force estimation have mostly been overlooked.

Here, motivated by the biological significance and potential broad implications for cell mechanics quantification, we combined computer simulations and analysis of experimentally acquired traction force data to establish the sensitivity of the TFM method to the Poisson’s ratio, and thus unravel how forces measured by TFM fundamentally rely on the correct value of the Poisson’s ratio.

## Results and discussion

### Conceptual interpretation of the influence of Poisson’s ratio in TFM

**Fig. 1:**
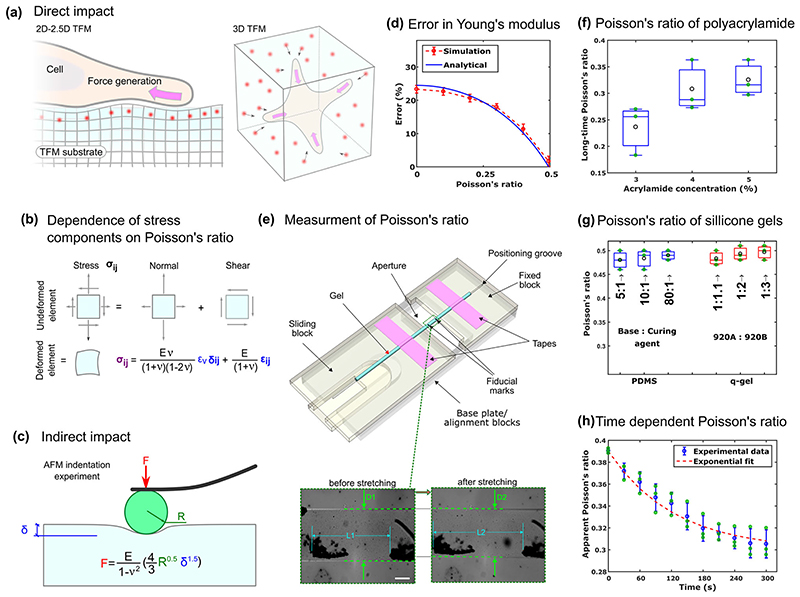
Conceptual analysis of the role of Poisson’s ratio. **a** Direct impact on stresses estimated by common traction force microscopy (TFM) modalities shown in the schematics. In 2D or 2.5D setting a cell adheres to the surface of a substrate and in the 3D condition a cell is embedded within a gel. **b** Cell generated forces deform a representative element in the vicinity of the cell. The normal and shear stress components are linked with the deformations through an isotropic linear elastic constitutive law that depends on Young’s modulus and Poisson’s ratio. **c** Indirect impact on estimation of the Young’s modulus measured via indentation tests. Fitting of the force-indentation curve with the simplest analytical Hertz model quantifies contact modulus: *E*/(1 – *ν* ^2^) rather than Young’s elastic modulus *E*. **d** Typically a Poisson’s ratio of 0.5 (incompressible material assumption) is considered to estimate the Young’s elastic modulus but this may generate some errors as the true values of the materials Poisson’s ratio deviates further from 0.5. The errors are obtained by considering either the realistic finite element (FE) simulations of the indentation (red line) or Hertz formulation (blue line). Error bars represent standard deviation (*n* = 15 simulations). **e** Schematic representation of the aligner device used to measure the Poisson’s ratio. Lower panels show images of a Polyacrylamide hydrogel (PAH) before and after stretching. When stretching, the distances in the direction of the stretch increases, while the diameter of the gel decreases. Scale = 250 μm. **f** Long-time Poisson’s ratio of PAH as a function of acrylamide concentration. The black circles show average and centre line represent median (n = 3 independent experiments). **g** Poisson’s ratio of q-gel and Polydimethylsiloxane (PDMS) for a range of elastomers proportions (n = 3 independent experiments). **h** Experimental demonstration of time-dependency of the Poisson’s ratio for PAH; Presence of large amount of liquid within hydrogels and the possibility of redistribution of the liquid within their pores make their mechanical responses time dependents. The term “apparent” implies that the time dependent changes observed in the diameter of the hydrogel is due to the liquid redistribution within the pores of hydrogel. Error bars represent standard deviation (n = 3 independent experiments).

To highlight the conceptional influence of the Poisson’s ratio we consider two typical TFM modalities (Fig. 1a); (i) when a cell is adhered to a substrate surface and (ii) when a cell is embedded in a three-dimensional (3D) gel matrix. In case (i), although the geometry is planar, the traction forces can be 3D in nature with normal and shear stresses deforming the substrate axially and laterally. The presence of both lateral and axial deformations on a planar substrate is commonly referred to as 2.5D-TFM while early 2D-TFM works^21,31^were only able to capture two-dimensional (2D) lateral displacements of the fiducial markers. In case (ii), referred to as 3D-TFM, the fluorescent markers are scattered throughout the bulk of the gel and both the geometry and the traction forces are 3D. In all cases, the cells apply forces to their adjacent environment, which are balanced by the stresses generated within the material located in the vicinity of the cell (Fig. 1b). The surrounding material in most TFM analysis, as well as this work, is considered to behave as a linear elastic material. The constitutive law for such materials indicates the dependence of both shear and normal stresses on the Poisson’s ratio in addition to the Young’s modulus (Fig. 1b). Therefore, any mismatch between the assumed Poisson’s ratio and the true underlying material Poisson’s ratio can lead to errors in the estimation of shear and normal stresses (Supplementary note 1). Additionally, considering the nature of the constitutive law, higher levels of error in the normal stress compared to those in the shear stress are expected (Supplementary Fig. S1 and Supplementary note 1). Specifically, the incompressibility assumption (*ν* = 0.5) may result in large errors in the normal stresses.

Beyond the direct involvement of the Poisson’s ratio in the force calculations, its misestimation during mechanical characterisation of the TFM substrate material can influence the measurements of the Young’s modulus, which is the most fundamental parameter in TFM analysis. Indeed, most mechanical characterisation techniques cannot estimate the Young’s modulus independently of the Poisson’s ratio. For example, AFM indentation, as one of the most common techniques used for the characterisation of soft substrates and hydrogels^32,33^, involves the application of defined forces and probing of the concomitant indentation displacements (Fig. 1c. To estimate the Young’s modulus, the resultant force-indentation curve is typically fit by a contact mechanics model such as the Hertz model (Fig. 1c), which is the simplest analytical formulation^34^ describing the contact behaviour observed in an AFM experiment. The outcome of these fitting procedures, in the case of the Hertz model, is the contact modulus *E*/(1 – *ν* ^2^) and thus calculation of the Young’s modulus (*E*), involves presuming a value for the Poisson’s ratio. Consequently, obtaining an accurate Young’s modulus is of utmost importance and requires precise determination of the Poisson’s ratio through an independent mechanical test (Fig. 1d).

Taken together, while achieving sufficiently high bead densities (BD) is a key determinant of TFM accuracy^16^, the intrinsic dependence of the stress and indirect reliance of Young’s modulus on the Poisson’s ratio suggest that even with perfect quantification of the substrate displacements, the predominate source of error in TFM could result from misestimation of this material property (Supplementary Fig. S1).

### Experimental quantification of the Poisson’s ratio of TFM substrates

Given the reliance of both stress estimation and mechanical characterisation, we developed a straightforward method of experimentally quantifying the Poisson’s ratio of substrates commonly used for TFM, including the polyacrylamide hydrogel (PAH), and two silicone gels, polydimethylsiloxane (PDMS) and q-gel (see “Methods” section). Several methods have been proposed to quantify the Poisson’s ratio of soft materials^26,35–37^ such as simple stretching of a long strip of material^18,38,39^, or more complex indention procedures^40^. In line with previously established methods, a strip of the gel was firmly attached to a simple aligner device consisting of a sliding block and a fixed component. Through an aperture in the device, the distance between fiducial markers manually placed on the gel was imaged using an optical microscope (Fig. 1e). By pulling the sliding block relative to the fixed part, the strip could be stretched leading to an increase in the lateral distance between the two fiducial markers (*L*_1_ < *L*_2_) and a decrease in the width (*D*_1_ > *D*_2_, Fig. 1e). Measurement of these geometrical changes allowed for the estimation of the Poisson’s ratio (see “Methods” section).

We measured the Poisson’s ratio of PAHs made with a range of acrylamide concentrations: 3, 4, and 5% which correspond to Young’s moduli of 1, 2, and 3 kPa, respectively. The Poisson’s ratio was larger for the PAHs with a higher concentration of acrylamide and it increased from 0.24 through 0.30 to 0.32 when the acrylamide concentration increased from 3% through 4 to 5% (Fig. 1f). In contrast to the PAH, the measured Poisson’s ratio of the two silicone gels (PDMS and q-gel) was close to 0.5 indicating a relatively incompressible behaviour for these gels. Additionally, varying the proportion of elastomers did not influence the Poisson’s ratio significantly for both gels (*p*-value > 0.2, Fig. 1g). Examination of the error bars in Fig. 1f, g showed that our method can quantify the Poison’s ratio with a good precision (±0.05) and the estimated values of the Poison’s ratio are in agreement with the values reported previously^22,36,40^. Nevertheless, a wide range has been reported for the PAH’s Poisson’s ratio as different methodologies, timescales, and concentrations of bis-acrylamide and ammonium persulfate were used in different studies^24,26^.

In order to maintain the level of hydration of the PAH during stretching and to replicate conditions in a typical TFM experiment, the PAH strip was submerged in PBS while attached to the device, and sufficient time was given for it to swell before stretching. Following fast stretching of a submerged PAH strip, the Poisson’s ratio was measured over time (Fig. 1h). After the initial stretch, the Poisson’s ratio values decreased from 0.39 to an asymptotic value of 0.30 after ~300 s. This time dependent behaviour can be explained through the poroelastic characteristic of the PAHs as discussed in refs. ^41–43^. The time-dependency of Poisson’s ratio in PAHs is in contrast with the behaviour of the silicone gels, whose Poisson’s ratio appeared to remain constant and nearly incompressible over time. While the Poisson’s ratio of the PAHs and other hydrogels is time dependent, this dependence is only significance when the timescale of the mechanical events is in the order of *L* ^2^/*D*_p_, where *L* is the involved length scale and *D_p_* is the poroelastic diffusion coefficient^44,45^. Within the context of TFM, considering that the length scale is smaller than the single cell size (*L* < 20 μm), the Poisson’s ratio approaches its asymptotic value within a fraction of a second for PAHs (with *D_p_* ~6 × 10^-9^ m^2^ s^-1^), while normally the cellular force generation occurs during 1–10 s for the fastest cellular processes (Supplementary note 7). This implies that asymptotic Poisson’s ratio would provide the most relevant value to be used in a linear elastic TFM analysis. Therefore, the values reported in Fig. 1f are the long-time (asymptotic) values of PAH’s Poisson’s ratios. Nevertheless, a poroelastic model describes the mechanical behaviour of the hydrogel more precisely, though carrying out such a complex analysis may not be computationally efficient.

### Assessment of influence of Poisson’s ratio in different TFM scenarios

**Fig. 2:**
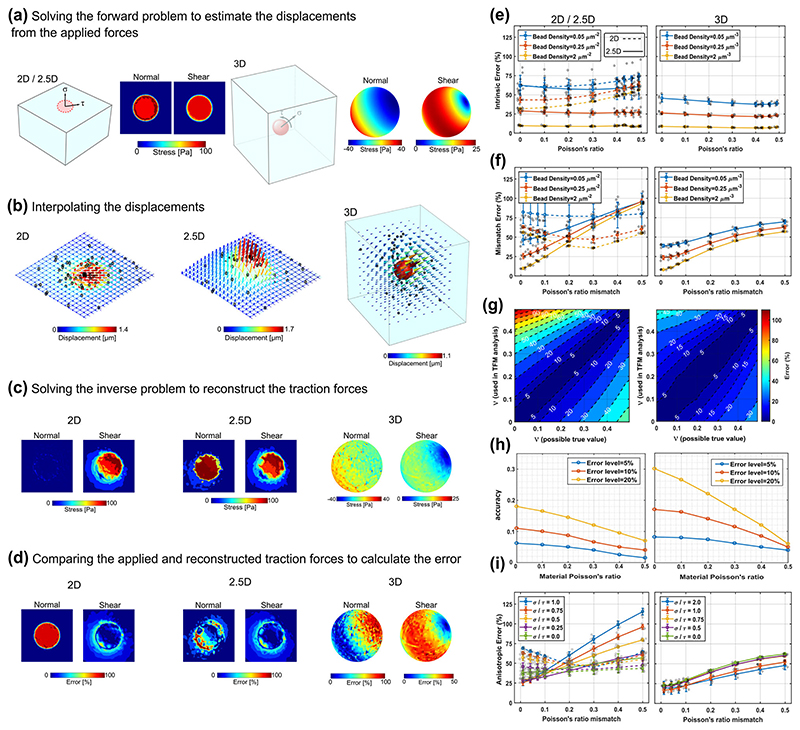
Assessment of the impact of the Poisson’s ratio on the accuracy of traction force microscopy (TFM) through finite element (FE) simulations. **a**-**d** Steps to compute error in the traction force estimation. **a** In 2D and 2.5D-TFM, shear and normal stresses are applied on a circular region located at the top surface of a cubic region with zero displacement boundary condition at the bottom surface. For 3D-TFM, a spherical traction region is defined within a cubic region whose deformation is again constrained at the bottom surface. Considering the linear constitutive law, the deformations resulting from the applied stress fields were calculated at all nodes within the FE mesh using the so-called forward Poisson’s ratio *ν* = *ν*_forward_, which represents the true Poisson’s ratio of the material. **b** The displacements were selected at random nodes (representing the finite sampling imposed by a given BD in a TFM experiment) and interpolated on the whole domain to obtain the displacement field. For the 2D case only lateral components of the displacement are extracted, while the axial component is discarded, replicating the lack of axial sensitivity in 2D-TFM. For 2.5D, both lateral and axial components of displacements are sampled from nodes at the top surface of the elastic substrate representing the typical experimental condition in 2.5D-TFM. To replicate 3D-TFM experiments, all components of the displacement from nodes throughout the cubic region were sampled. **c** The interpolated displacements were used to solve the inverse problem considering a *ν* = *ν*_inverse_, which is a Poisson’s ratio that is hypothetically assumed to reconstruct the forces in typical TFM analysis. **d** The initially applied 3D traction forces were compared with the reconstructed ones to find the error. **e** Even considering the ideal case of *ν*_forward_ = *ν*_inverse_, some intrinsic errors are generated which are highly dependent on the sampling density. 2D and 2.5D results are represented with dash and solid curves, respectively (n = 10 for 2D/2.5D and n= 5 for 3D simulations). **f** The mismatch error was estimated considering *ν*_inverse_ = 0.5 and *ν*_forward_ to vary from 0 to 0.5 generation Poisson’s ratio mismatch (*ν*_inverse_ – *ν*_forward_) ranging from 0.5 to 0 (n = 8 for 2D/2.5D and n = 5 for 3D simulations). **g** Contour maps showing the errors generated as the result of considering all combinations of *ν*_forward_ (possible true value) and *ν*_inverse_ (used in TFM analysis) varying from 0 to 0.5 for the 2.5D (left) and 3D (right) scenarios. **h** Bilateral tolerance in the Poisson’s ratio for three allowable levels of traction error for the 2.5D (left) and 3D (right) scenarios. **i** Effects of force anisotropy on the error. To generate different force anisotropy, the ratio of normal to shear stress was varied from 0 to 1 for 2D and 2.5D cases and from 0 to 2 for the 3D case. (n = 5 simulations). Error bars represent standard deviation in all panels.

Having measured the typical range of Poisson’s ratio for materials commonly used for TFM, we next assessed its potential impact on TFM measurements via computer simulations in representative 2D, 2.5D, and 3D-TFM scenarios (Fig. 2a–d). First, a specified stress field was applied within a defined region and the concomitant displacements in the whole domain were estimated by finite element (FE) method considering a specified Poisson’s ratio (forward problem) (Fig. 2a). To mimic TFM experiments, displacements were sampled at several random points (representing a defined BD) relevant to each scenario (Fig. 2b). The sampled displacements were then interpolated to estimate the strain field and considering a presumed Poisson’s ratio the FE simulations were run again to estimate the stress field (inverse problem) (Fig. 2c). Finally, the error was calculated by comparing the simulated (forward problem) and reconstructed (inverse problem) stresses (Fig. 2d).

When the Poisson’s ratio is matched between the forward and the inverse problems (i.e., the Poisson’s ratio of the material is precisely known), the error was found to be mainly dependent on the sampling density, while a weak dependence on the value of the Poisson’s ratio was observed, specifically, in the 2.5D scenario, regardless of the value of Poisson’s ratio, the magnitude of error was 59, 27, and 9% for BD = 0.05, 0.25, and 2 μm^−2^, respectively. As it has been investigated by other researchers^16,46^, for lower BDs, such a significant increase in the error (p-value < 0.0001) supports the application of super-resolution-based TFM to sample high BDs and accurately localise the position of the fiducial markers (Fig. 2e and Supplementary Note 1). We also investigated the impact of BD for the 3D scenario which involves interpolation of displacements throughout the whole domain. The 3D scenario exhibited slightly lower levels of error (Supplementary Note 3), with the error falling to 40, 23, and 8% for BD = 0.05, 0.25, and 2 μm^−3^, respectively.

Next, the level of the error resulting from a Poisson’s ratio mismatch were evaluated by considering the actual Poisson’s ratio of the material (in the forward problem) to vary from 0 to 0.5, while in the inverse problem the Poisson’s ratio was maintained at 0.5 which is a value that has been assumed in many previously published TFM works^15,47–60^ (leading to mismatches from 0.5 to 0, Fig. 2f). Strikingly, as the mismatch increases from 0 to 0.5, the error also increases monotonically from 10 to 93% in the 2.5D scenario (BD = 2 μm^−2^) and from 8 to 57% in the 3D scenario (BD = 2 μm^−3^). For 2.5D scenario, the curves corresponding BD = 0.05, 0.25, and 2 μm^−2^ converge as the mismatch approaches 0.5. For the 2D scenario the relationship between the error and the mismatch is biphasic (see Supplementary Note 2 for further discussion). When no Poisson’s ratio mismatch exists between forward and inverse problems (Fig. 2e), the error curves corresponding to BD = 0.05, 0.25, and 2 μm^−2^ indicate the isolated impact of BD. On the other hand, Fig. 2f displays the combined effects of BD and Poisson’s ratio mismatch on the errors. For example, in the 2.5D scenario, when BD = 2 μm^−2^, up to 10% errors can be expected considering only the effects of BD (Fig. 2e), while the combined effects of BD and Poisson’s ratio mismatch lead to 93% error for a mismatch of 0.5 (Fig. 2f). Similarly, in the 3D scenario and for BD = 2 μm^−3^, the error increases from ~8 to 57% when the Poisson’s ratio mismatch of 0.5 is considered. Such dramatic increases in the errors demonstrates the importance of considering the Poisson’s ratio alongside BD for accurate reconstruction of traction forces. It is worth mentioning that although the experimental data suggest that the PAHs have a Poisson’s ratio in the range of [0.24–0.4] (Fig. 1f) and both silicone gels exhibit a nearly incompressible behaviour (Fig. 1g), in Fig. 2, the Poisson’s ratio was varied in the full range of [0–0.5] to examine all the conditions that a biomaterial may potentially exhibit.

To generalise our conclusions and present experimentalist with a practical guide, we obtained a contour map of the error by estimating the error for any combination of Poisson’s ratio in the forward (materials actual value) and inverse (considered in TFM analysis) problems (Fig. 2g). Considering the iso-lines in the contour map, it is possible to predict the degree of accuracy required in the measurement of the Poisson’s ratio to maintain the errors below a specific level (Fig. 2h). For example, in 2.5D-TFM for a material with a true Poisson’s ratio of *ν* = 0.4, to keep the overall error below 5%, the Poisson’s ratio used in the TFM analysis must be known to the accuracy level of *ν* ± 0.02 (i.e., 0.4 ± 0.02).

Lastly, we assessed the role of force directionality and how the Poisson’s ratio mismatch influences the estimation of shear and compressive/tensional forces (Fig. 2i) by changing the ratio of the normal (*σ*) to shear (*τ*) stress from 0 (presence of only shear forces) to 1 (equal shear and normal forces). As expected, in the 2D scenario the anisotropy in the forces has a negligible effect since the normal component is excluded. In contrast, in the 2.5D scenario, the error increases when either the mismatch in Poisson’s ratio increases for a given ratio *σ*/*τ* or the ratio *σ*/*τ* increases for a given mismatch in Poisson’s ratio (Fig. 2i). Importantly, when *σ*/*τ* is greater than ~0.1 (which corresponds to displacement ratio *U*_axial_/*U*_lateral_ > 0.06) the difference of the error between 2.5D and 2D is considerable, meaning, in such circumstances, ignoring the axial displacement can lead to significantly large errors (*p*-value < 0.05, Supplementary Fig S10b and Supplementary Note 4) and thus tracking fluorescent markers in all three directions is essential to ensure accurate force reconstruction. Additionally, for 3D-TFM the effect of stress anisotropy is not significant while for 2.5D the reconstructed stresses are highly sensitive to the axial displacements (Supplementary Fig. S10d and Supplementary Note 4), which further corroborates the importance of accurate measurement of the axial displacements.

### Experimental assessment of the influence of Poisson’s ratio in TFM

**Fig. 3:**
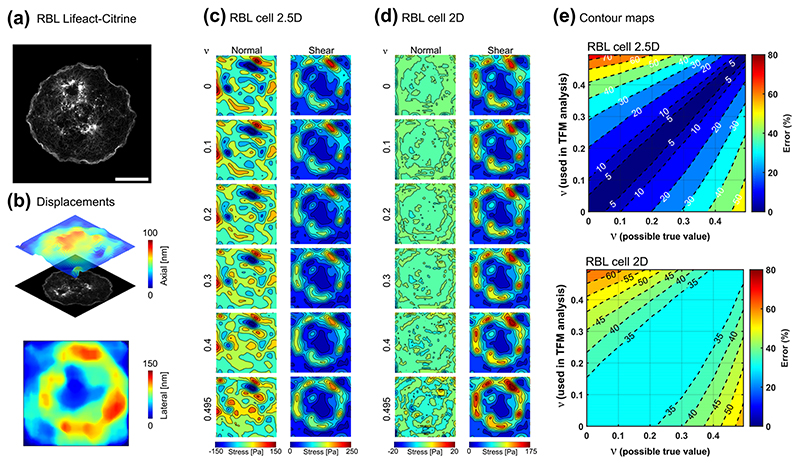
Impact of Poisson’s ratio on the experimentally acquired data from a rat basophilic leukaemia (RBL) cell. **a** Total internal reflection fluorescence structured illumination microscopy (TIRF-SIM) image of an RBL cell expressing Lifeact-citrine activating on an antigen coated elastic substrate. Scale = 5 μm. (reproduced and reanalysed data from Li et al.^61^) **b** The axial and lateral displacements during RBL cell activation were estimated by employing astigmatic traction force microscopy (aTFM). **c** Magnitude of the reconstructed normal and shear stresses considering both axial and lateral components of the displacement when the Poisson’s ratio of the substrate ranges between 0-0.5. **d** Magnitude of the reconstructed normal and shear stresses when the axial component of displacements was ignored and only the lateral components were considered. **e** Contour map of the error for a hypothetical problem (for RBL cell activation) considering all combinations of the possible true Poisson’s ratio and the Poisson’s ratio used in TFM analysis, varying from 0 to 0.5.

**Fig. 4:**
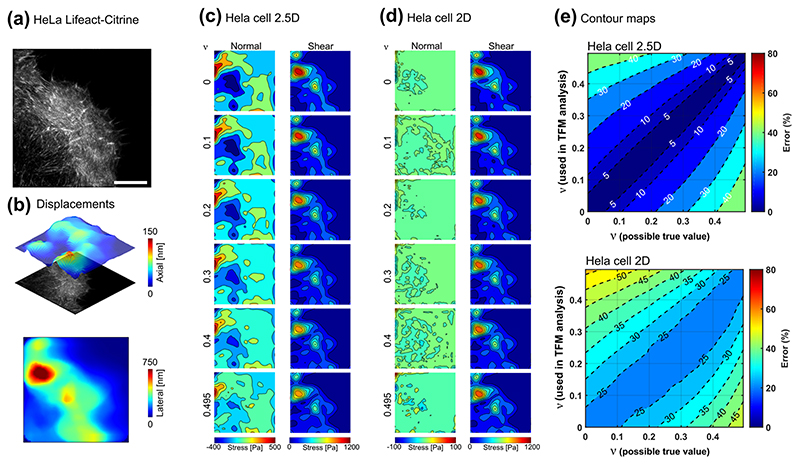
Impact of Poisson’s ratio on the experimentally acquired data from a HeLa cell. **a** Total internal reflection fluorescence structured illumination microscopy (TIRF-SIM) image of a HeLa cell adhering to the elastic substrate. Scale = 5 μm (reproduced and reanalysed data from Li et al.^61^). **b** Magnitude of axial and lateral displacements during HeLa cell adhesion were estimated by employing astigmatic traction force microscopy (aTFM). **c** Magnitude of the reconstructed normal and shear stresses considering both axial and lateral components of the displacement when the Poisson’s ratio of the substrate ranges between 0-0.5. **d** Magnitude of the reconstructed normal and shear stresses when the axial component of displacements was ignored and only the lateral components were considered. **e** Contour map of the error for a hypothetical problem (for HeLa cell adhesion) considering all combinations of the possible true Poisson’s ratio and the Poisson’s ratio used in TFM analysis, varying from 0 to 0.5.

Having simulated the extent to which the accuracy of the Poisson’s ratio affects the traction reconstruction in 2D, 2.5D, and 3D-TFM scenarios, we next investigated how our findings extend to real experimental settings. In contrast to the methodology of Fig. 2, in which a uniform load was applied on a circular region, in this section we utilise experimentally acquired cell geometry and cell-induced displacement fields. This allows us to investigate the relevance of the idealised results shown in Fig. 2 to real TFM experiments. We focus on investigating 2.5D-TFM data, acquired using astigmatic-TFM (aTFM)^61^, since the simulation results demonstrated that the impact of Poisson’s ratio mismatch is more evident in the 2.5D scenario compared to the 3D condition (Fig. 2e, f, i). Immune cell activation and cell adhesions exhibit shear and normal stresses when in contact with the TFM substrate, making them interesting biological systems within which the effects of the materials Poisson’s ratio on the resulting force distribution could be studied. In the first example, a Rat Basophilic Leukaemia (RBL) cell is activated on an antigen coated surface while generating shear and normal forces, leading to lateral and axial deformations (on the 100-150 nm length scale) of the substrate (Fig. 3a, b). For a typical 2.5D-TFM scenario both axial and lateral displacements were implemented (Fig. 3c) while to mimic the 2D scenario the axial component was ignored (Fig. 3d). Assuming a range of values for the Poisson’s ratio led to different distribution of reconstructed normal and shear stresses in each scenario. The q-gel substrate displayed an almost incompressible behaviour (*ν* ≈ 0.5 as measured in Fig. 1g), and therefore considering the incompressibility assumption in the simulations yields a negligible mismatch error. Nevertheless, if we consider a hypothetical case with a PAH (with 3% acrylamide and a Poisson’s ratio of 0.24 as measured in Fig. 1f) as the TFM substrate and displacement field is exactly similar to those measured in our TFM experiments, the incompressibility assumption in the TFM analysis can lead to 55% error for the RBL cell (Fig. 3e). Contour maps in Fig. 3e, display estimated errors for any combination of possible ground truth Poisson’s ratio (possible true value) and the ratio used in the simulations (used in TFM analysis). Similar analyses were conducted for a HeLa cell undergoing early substrate adhesion (Fig. 4a-e). Importantly, when the axial component of displacement is ignored (which is the 2D-TFM scenario), a minimum of ~35% error for immune cell activation and ~25% for cancer cell adhesion was observed even in absence of any mismatch in the Poisson’s ratio. The error is larger for the RBL cell which exhibits a higher ratio of normal to shear stresses as predicted by our analysis as in Fig. 2i. Additionally, for a 2.5D scenario, a mismatch of 0.2 in the Poisson’s ratio leads to up to 50 and 30% error in constructed tractions for the RBL cell and HeLa cell, respectively (Figs. 3e and 4e). Taken together, the errors for both cell types are in close agreement with the plots of Fig. 2 and the error contours indicate less sensitivity of forces to the Poisson’s ratio for the HeLa cell adhesion compared to RBL cell activation.

Beyond the effects of Poisson’s ratio mismatch, displacement noise is another source of uncertainty, which may generate significant errors^46^. Empirically, the applied TFM modality using fast single-frame astigmatic imaging coupled with total internal reflection fluorescence microscopy has a minimum experimental lateral and axial displacement uncertainty of approximately 4 and 7 nm^61^, respectively (Supplementary note 6). This leads to errors of ~6 and 17 Pa in shear and normal stresses, respectively (Supplementary Fig. S12), which are insignificant compared to the magnitude of the traction stresses generated by RBL and HeLa cells.

## Conclusions

Quantifying the mechanical forces generated by living systems is key to furthering the understanding of biomechanics and mechanobiology. Hence, building tools that can reliably quantify these forces at sufficient sensitivity without perturbing the biological system is essential. Together with advances in high-resolution optical imaging, TFM represents a powerful methodology to address these requirements. Nevertheless, the sensitivity of TFM is fundamentally reliant on knowledge of the material’s Young’s modulus and the Poisson’s ratio. In this work, we developed a framework for determining the error levels introduced via misestimation of the Poisson’s ratio in TFM, demonstrating the critical importance of using the correct material Poisson’s ratio by simulating common TFM modalities and evaluating experimentally acquired data.

In the current study, reliable quantification of the long-time Poisson’s ratio was acquired through development of a simple platform, which enabled us to calculate the Poisson’s ratio directly without any specific assumption. Additionally, to quantify the Poisson’s ratio, an optimum length-scale of ~1mm was adopted for the gel samples. This length scale is sufficiently large that allows the precise measurement of displacements using conventional light microscopy while it is small enough that the samples reach their long-time (asymptotic) state within a reasonably short timescale. Employing the platform, we demonstrated that silicone gels display a nearly incompressible and time-independent behaviour irrespective of their elastomer proportions. On the other hand, the Poisson’s ratio of PAH is considerably less than 0.5 and a function of the PAH constituent concentrations. Furthermore, since PAHs exhibit poroelastic behaviour, their Poisson’s ratio is time dependent and converges to an asymptotic value at long time scales, which is a fraction of a second within the context of TFM. The different mechanical behaviour of these gels highlights the potential challenge for the selection of the substrate material for use in TFM experiments and setting material values in the TFM analysis. Future investigation of the mechanical behaviour of the substrates beyond a linear elastic model and considering the time-dependent (such as viscoelasticity and poroelasticity) behaviour will be of critical importance towards a more accurate and robust quantification of cell generated forces.

Our analyses lead to several important experimental considerations. Firstly, consistent with previous works^16^ we have reevaluated that the density of the fiducial markers is a key parameter in determining the overall accuracy of the TFM in all geometries in 2D, 2.5D, and 3D. In addition to the bead density, we found that small errors in the estimation of fiducial marker location can lead to large errors in traction reconstruction (for example location errors in the order of 2% can generate traction errors of 25%, Supplementary Fig. S1). These results further emphasise the importance of using an imaging modality with a spatial resolution sufficient to capture mechanical details within a given biological system.

Secondly, we demonstrated that the Poisson’s ratio is of particular importance in 2.5D and 3D TFM scenarios, where both lateral and axial substrate deformations are present, with a mismatch of 0.2 in the Poisson’s ratio leading to errors of up to 100% for both the magnitude and distribution of constructed traction (particularly the axial component) (Supplementary Figs. S6–S8). This key finding suggests that when the ratio of the normal and shear stress is greater than ~0.1, the axial components of displacement cannot be ignored and the application of 2D TFM may result in incorrect reconstruction of forces (Supplementary Fig. S9). With the increasing availability of techniques that can quantify both 2.5D and 3D displacements^25,29^, these results will serve as a key guide for experimentalist in choosing the appropriate TFM modality to address their biological question.

Finally, to frame our conclusions into a wider practical perspective, we introduce a robust measure of the sensitivity of TFM to the Poisson’s ratio by analysing the error in the traction force reconstruction across a wide range of Poisson’s ratios (Fig. 2h). We provide a quantitative method and guide demonstrating the accuracy required in the measurement of the Poisson’s ratio to achieve a desired level of error. These results will inform both the future characterisation tools (for more accurate quantification of the material properties^62^), as well as TFM experimental methods and analysis techniques. In addition, our work highlights the importance of establishing the correct value of the Poisson’s ratio not only in TFM measurements but more broadly to any cell mechanics measurements relying on a linear elastic framework.

## Methods

### Device fabrication

The Poisson’s ration measurement device was fabricated by cutting (Epilog laser cutter) the design through 2mm thickness polymethyl methacrylate (PMMA) sheets. It comprised three layers sitting on top of each other. The size of the device is 62 × 25 × 6 mm in an unstretched state. The two lower blocks (baseplate/alignment blocks in Fig. 1e) and the fixed block were bonded together by chemical bonding (SciGrip Weld ON 4SC). A 20 × 10mm aperture on the lower blocks enabled microscope imaging. The moving block, in turn, consisted of three layers, bonded together using the adhesive. The two lower layers fit tightly inside slits on the fixed block, constraining the sliding block to move forward/backward only in one direction.

### Gel preparation

To prepare PAH, acrylamide (with final concentrations of 3, 4, and 5%, Sigma), bis-acrylamide (0.1%, Severn Biotech), Ammonium persulfate (1%, Sigma), and TEMED (0.1%, Sigma) were dissolved in PBS. After mixing, around 40 μL of the solution was immediately directed into a tube (inner diameter 1 mm, Agilent Technologies) using a 1 mL syringe (BD plastic) connected to the other end of the tube and left there for 15 min at room temperature to cure. Then, the gel was expelled from the tube gently and at the same time was placed on the stretching device.

To prepare q-gel, parts A and B of q-Gel 920 (Quantum Silicones LLC), were mixed with ratios of 1:1.1, 1:2, and 1:3 and were transferred into a 30 × 3 × 3mm mould. After degassing the mixture, it was baked at 100 °C for 90min. Then the cured gel was removed from the mould carefully and mounted on the device. To prepare PDMS, the elastomer and curing agent (Ellsworth adhesives) were mixed with a ratio of 80:1, 10:1, and 5:1, and the mixture was moulded and degassed followed by baking at 90 °C for 90min. For the measurements, a very thin (~1mm) layer of the PDMS were peeled off from the bulk and placed on the stretching device.

### Poisson’s ratio measurement

The strips of different gels were placed carefully on the stretcher device and taped firmly to the fixed and sliding blocks. To ensure that the strip lied straight, it was placed on the positioning grooves (Fig. 1e). As fiducial markers, two stains were inked on the strip using a permanent marker. For hydrogels, unlike silicone gels, the device was placed in a Petri dish filled with PBS and the hydrogel was allowed to swell for 2 h. Using an optical microscope (Leica DMi 8), the gel was imaged once, and after being stretched by pulling the sliding block, the imaging was continued every 30 s for 5 min.

To compute the Poisson’s ratio, sample’s diameter and also the distance between the two stains were measured at least at five different locations (*L*_1_, *L*_2_, *D*_1_, and *D*_2_ in Fig. 1e, in which subscripts 1 and 2 represent before and after starching of the gel, respectively). Axial and lateral strains were calculated using ε_a_ = (*L*_2_ - *L*_1_)/*L*_1_ and ε_l_ = (*D*_2_ - *D*_1_)/*D*_1_, respectively at each location and their averages were obtained. Finally, the Poisson’s ratio was calculated using $\nu =-{\bar{\varepsilon }}_{1}/\bar{\varepsilon }$ in which ${\bar{\varepsilon }}_{a}$ and ${\bar{\varepsilon }}_{1}$ represent the average axial and lateral strains, respectively. For statistical analysis, each experiment was repeated at least three times. The values reported in Fig. 1f, g are the long-timescale Poisson’s ratio (Supplementary note 7) that were obtained from the images taken 5 min after stretching the gel.

### Finite element simulations

The results presented in the main figures of this study were obtained from FE analyses for both direct and inverse problems. FE simulations provide straightforward and robust solutions and benefit from key features including the flexibility to simulate complicated geometries, the ability to model materials with different degrees of compressibility and more complex constitutive laws, as well as the capability to include nonlinear effects. Furthermore, for the inverse problem, the FE analysis may provide more robust solutions compared to Tikhonov regularised Green function’s methodology (Supplementary note 5). It is worth noting that FE uses the similar steps to solve the forward and inverse problems including: discretisation of the domain into finite elements, finding the stiffness matrix for each element (that only depends on the geometry and material properties of the element), assembly of the stiffness matrices of the elements to form the global stiffness matrix, applying natural boundary conditions to form the global load vector, applying the essential boundary conditions to the algebraic system of equations, solving the system of equations to obtain the displacement field and the secondary field variables such as stress. Thus, the only difference between the forward and inverse problem is in the type of boundary conditions applied to the algebraic system of equations. For the forward and inverse TFM problems, the discrete nature of FE method guarantees that the system of equations will turn into a well-posed problem after applying the boundary conditions and therefore no regularisation is required to solve the problem. FE simulations were carried out using a commercial FE software (ABAQUS, Dassault systems, France). Details of simulations and the parameter selections for solving the forward and inverse problems have been extensively described in ref. ^29^. Briefly, the domain size was considered large enough to minimise the finite domain boundary effects. Mesh size was adopted after carrying out a mesh sensitivity analysis. A quadratic tetrahedral 10-node element using hybrid formulation with improved surface stress visualisation (C3D10HS) was employed to discretise the domain. The isotropic linear elastic constitutive law with large deformation formulation, which captures geometrical nonlinearity was implemented. A comparison between FE method and the Tikhonov regularised Green’s function method is also provided in Supplementary Note 5.

To assess the influence of misestimation of the Poisson’s ratio on estimation of the Young’s modulus obtained from AFM (Fig. 1d), a FE model of a rigid sphere indenting a deformable substrate of original *E* and *ν*, was employed. The shear and normal behaviour of the contact was selected as rough and hard contact, respectively. The force-indentation curve obtained from the simulation was used to derive E/(1 - *ν* ^2^). Then, assuming *ν* = 0.5, the Young’s modulus was estimated, and the respective error was computed by comparing the estimated Young’s modulus with the original E. For each original *ν*, such procedure was repeated for several bead sizes, indentation depths and original Young’s modulus to find the average and standard deviation of the error (Fig. 1d).

### Cell culture

The experiments were performed using RBL-2H3 clone cells (CRL-2256, ATCC, USA; mycoplasma tested) and HeLa cells (product 93021013, Sigma-Aldrich; mycoplasma tested). Stable expression of Lifeact-citrine in both RBL and HeLa cells was achieved via a lentivirus transduction strategy. RBL cells were maintained at 37 °C in 5% CO_2_ in minimum essential media (MEM) (Sigma Aldrich) containing 15% fetal bovine serum (FBS), 10mM HEPES (Lonza, UK), 2 mM L-glutamine and 1% penicillin–streptomycin. HeLa cells were maintained at 37 °C in 5% CO_2_ in DMEM (Sigma-Aldrich) supplemented with 10% FBS, 2 mM L-glutamine and 1% penicillin–streptomycin. Cells were split every two days at a volume ratio of 1:5. Twenty-four hours prior to TFM experiments, RBL cells were treated with 0.05% Trypsin-EDTA (Lonza), facilitating their detachment from the cell culture flask. Cells were then transferred to a rotating chamber at 37 °C in 5% CO_2_ to maintain their suspension state prior to experiments.

### Astigmatic TFM (aTFM) measurements

The data presented in Figs. 3 and 4 was acquired using astigmatic TFM conducted on RBL and HeLa cell lines. By combining super-resolved TIRF-SIM microscopy with astigmatic imaging, aTFM is able to capture nano-scale axial and lateral deformations of the underlying gel substrate, in addition to a super-resolved SIM image of the cell in contact with the substrate. Full details of the experimental methods of aTFM are provided in ref. ^61^.

### Statistics

For aTFM experiments on both HeLa and RBL cells, force data was acquired in at least 20 cells, from three independent experiments, in each case of which representative data is presented in Figs. 3 and 4. Random node sampling was repeated more than five times to obtain each data points in Fig. 2e, f, i. Error bars represent standard deviation and a two-tailed *t*-test was carried out to compare the errors and compute *p*-values.

## Supplementary Material

Supplementary Information

## Data Availability

The data that support the findings of this study are available from the corresponding authors upon reasonable request.

## Reporting summary

Further information on research design is available in the Nature Research Reporting Summary linked to this article.
