# Supplementary material for: Quantifying cell-generated forces: Poisson’s ratio matters
Source: Commun Phys. Author manuscript; Available in PMC 2021 Nov 26. (PMC7612038; doi:10.1038/s42005-021-00740-y)
Supplement: Supplementary Information [file EMS138996-supplement-Supplementary_Information.pdf]

Supplementary Information: Quantifying cell-generated forces: Poisson's ratio matters

Yousef Javanmardi[1#], Huw Colin-York[2#], Nicolas Szita[3], Marco Fritzsche[2,4*], Emad Moeendarbary[1,5*]

[1]Department of Mechanical Engineering, University College London, London, WC1E 7JE, UK.

[2]Kennedy Institute for Rheumatology, Roosevelt Drive, University of Oxford, Oxford, OX3 7LF, United Kingdom.

[3]Department of Biochemical Engineering, University College London, London, WC1E 6BT, UK.

[4]Rosalind Franklin Institute, Harwell Campus, Didcot, OX11 0FA, United Kingdom.

[5]Department of Biological Engineering, Massachusetts Institute of Technology, Cambridge, Massachusetts, USA.

*Correspondence to:
Marco Fritzsche: marco.fritzsche@kennedy.ox.ac.uk
Emad Moeendarbary: e.moeendarbary@ucl.ac.uk

# These authors contributed equally

Description of Supplementary notes

In the following, we present seven supplementary notes to support the information provided in the manuscript:

In **note 1** and **2**, using Timoshenko beam theory, an analytical approach is adopted to solve a simplified TFM problem. Such an approach would provide insightful information on the separate role of bead density and Poisson's ratio on reconstructed shear and normal tractions and helps understanding the biphasic behaviour observed in **Fig. 2e** and **2f** obtained from FE analysis for the 2D scenario. **Note 3** depicts the distribution of normal and shear tractions applied to the substrate for the 2D, 2.5D, and 3D scenarios, and compares them with the reconstructed tractions obtained from FE analysis. Sensitivity of normal and shear tractions with respect to axial and lateral components of displacements are evaluated in **Note 4** using FE simulations. In **Note 5**, the results obtained from Green functions method are compared with those obtained from FEM and the differences observed for forward and inverse problems are discussed in detail. Employing FEM, **note 6** assesses the uncertainties involved in determining the reconstructed tractions due to noise in the measurement of axial and lateral displacements. In **note 7**, we show that why using asymptotic Poisson's ratio provides the most relevant representative value to be used for TFM traction recovery computations.

Supplementary Note 1- Impact of Bead density and Poisson's ratio on shear and normal stress: an analytical investigation for an ideal problem

In the current study, we aim to investigate the impact of Poisson's ratio mismatch on the accuracy of recovered traction forces. However, in a TFM experiment sampling error always exists since the imaging resolution is limited and therefore only tracking of a certain density of the fluorescent markers is possible. Therefore, in the main text the combined impact of these two factors were examined. However, in this note, we investigate the errors arising from bead density and the Poisson's ratio independently. To this end, we consider an ideal problem for which an analytical solution is available, and thus, the impact of each factor could be determined theoretically. To this end, we consider a cubic substrate to which uniform shear and normal stresses are applied to its top face while the bottom face is constrained in three directions (Fig. S1.a). The magnitude of displacements on the top face is obtained by solving the so called forward problem analytically, converting a known stress field into a displacement filed, assuming $\nu_{forward} = 0$ (Fig. S1.b). Spatial points are chosen at random in the domain, representing the random location of the fiducial markers in TFM, and their displacements recorded. Then, using interpolation, similar to conventional TFM, the displacement field is derived within the entire domain (Fig. S1.b). Comparing the interpolated displacement with the analytical one, gives the histogram of displacement error for each BD. Fig. S1.c shows that BD=0.05, 0.25, and 2.00 μm^{-3} generate relative errors in the displacements between 2 and 0%. Finally, the inverse problem is solved whereby the traction forces can be reconstructed from the strains using Eq. S4. Here, to replicate a TFM problem with an incompressible substrate, we assume $\nu_{invesre} = 0.5$. Analytical normal and shear stresses are compared with the reconstructed stresses in Fig. S1.d and e, respectively and the histograms in Fig. S1.f show the difference between analytical (forward) and reconstructed (inverse) stresses. Crucially, Fig. S1.f demonstrates that even for BD = 2, where the displacement errors are negligible, there is a ~66% and ~33% error in normal and shear stresses, respectively. In addition to the importance of the bead density, this result emphasises the significance of accurately quantifying the positions of the fiducial markers, further supporting the application of super resolution microscopy in conjunction with TFM.

A similar procedure can be repeated whereby the assumed material Poisson's ratio differs from that applied during the TFM analysis, defining a so-called Poisson's ratio mismatch. Fig. S1.g details the error in normal and shear stresses as well as the average of the error for all components of the stress tensor for Poisson's ratio mismatches ranging from 0 to 0.5. The black dash-line in the plots show the analytical error due to Poisson's ratio mismatch alone when the displacement field is perfectly quantified. The plots confirm our considerations from Fig. S1.f, where an increase in BD results in a decrease in the mean and standard deviation of errors. In addition, the errors in normal stress are twice as large as errors in shear stress, indicating the key importance of the Poisson's ratio for the accurate reconstruction of normal forces.

As mentioned above, the black dash-curves in **Fig. S1g** were obtained from an analytical solution in which displacement is described as a continuous function. In such a case displacement is known at any arbitrary point, thus, bead density could be considered to be infinite. In other words, the black dash curves represent the errors due to only Poisson's ratio mismatch (where no sampling error exists). On the other hand, coloured solid curves in **Fig. S1g**, represent the errors from the combined effects of Poisson's ratio mismatch and sampling density. Therefore, the difference between the black curve and colour curves could be inferred as the errors due to only sampling density and careful examination of the curves in **Fig. S1g** suggests that such an error for all types of stresses is almost independent from Poisson's ratio mismatch, since the dashed-black and coloured curves are almost parallel. Two more important points could be deduced from **Fig. S1g**: 1) Sampling density error in normal and shear stresses is 35% and 7%, respectively, for a bead density of 0.05. This indicates that compared to shear stresses, normal stresses are more influenced by sampling density. 2) When sampling density is not sufficiently large, displacements are not estimated accurately, and this in turn causes misestimation of the constructed tractions. **Fig. S1c** shows that such errors in displacement is less than 2% for a bead density of 0.05. Such a small error in displacement generates errors as large as 35% and 7% in normal and shear stresses, respectively. This, in agreement with the findings of other researchers[1], demonstrates the importance of finding the position of beads precisely.

It should be noted that while the idealised problem studied in the current note cannot fully replicate a TFM problem, its results can help understanding the mechanisms controlling the magnitude of error in reconstructed stresses. Furthermore, the analytical method used for **Note 1** provides an instant clear picture of errors that are qualitatively consistent with the results obtained from the numerical FE methods used for **Fig. 2** which required extensive number of FEM simulations.

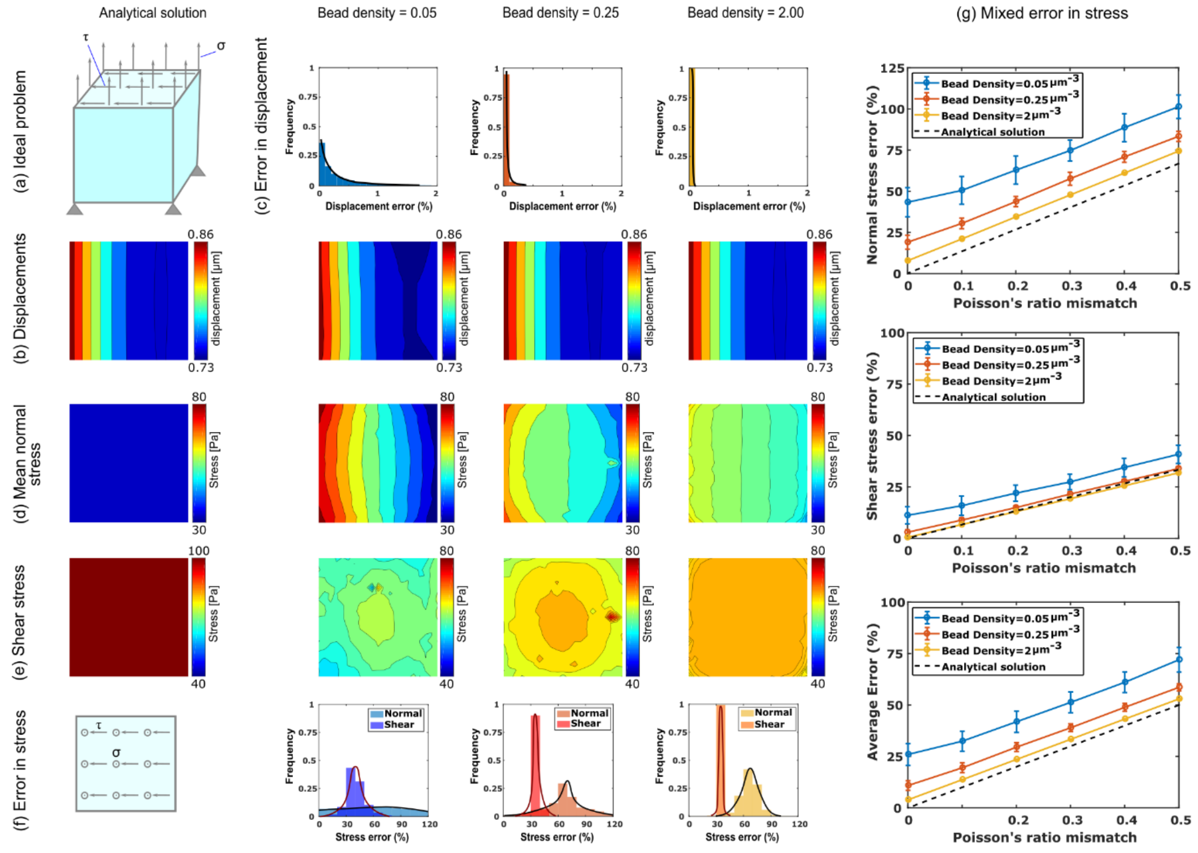

Supplementary Figure S1. *The impact of bead density and Poisson's ratio on the magnitude of error in shear and normal tractions.* ***(a)*** *Geometry and boundary conditions of an idealised problem for which analytical solution is available. The domain consists of a cube which its lower face is constrained in three direction while a normal (σ) and shear (τ) traction are being applied to the upper face. For the sake of simplicity, we assume $\sigma=\tau$.* ***(b)*** *Displacements on the top face of the substrate are found analytically by solving the forward problem (i.e. the problem of establishing the unknown displacements from the known traction forces). For the forward problem, the Poisson's ratio is assumed to be $\nu_{forward}=0$. Contours show norm of the displacements vector. Then, several nodes, corresponding to bead densities of 0.05, 0.25, and 2 μm^{-2}, are chosen randomly throughout the substrate and their displacements record. At the end of this step, the displacements are known at some discrete points and using interpolation/extrapolation, they can be found at any arbitrary node. The norm of displacement vector on top face of the substrate is also shown after interpolation/extrapolation for BD= 0.05, 0.25, and 2μm^{-2}.* ***(c)*** *Comparing the analytical displacement with the interpolated/extrapolated one at all points, the error is computed for each BD. The magnitude of error ranges between 0 and 2%, while lower bead densities correspond to larger errors.* ***(d, e)*** *Having established the displacements in the previous step, the traction forces can be computed by solving the inverse problem. For the inverse problem, the Poisson's ratio is assumed to be $\nu_{inverse}=0.5$. In other words, the displacements obtained in the previous step, both analytical and interpolated/extrapolated ones, are applied to the domain to reconstruct the normal and shear traction forces represented in part d and e, respectively.* ***(f)*** *Comparing the reconstructed normal and shear tractions for BD=0.05, 0.25, and 2 μm^{-2} with the exact analytical solution, the error in estimating of the traction forces is computed for each BD. These results demonstrate that the error in normal tractions is larger than that in shear stresses. Furthermore, for higher bead densities, the mean and standard deviation of the error would be less. Additionally, even for case BD=2 μm^{-2}, where the displacements are quantified almost perfectly, there would be ~66% and ~33% error in normal and shear tractions, respectively.* ***(g)*** *The procedure of solving the direct and inverse problem can be repeated for different values of $\nu_{forward}$ ranging from 0 to 0.5, while it is kept constant for the inverse problem, i.e. $\nu_{inverse}=0.5$ (incompressible material). This gives the error due to incompressibility assumption in TFM analysis as a function of the mismatch in Poisson's ratio which is defined as the difference between true underlying material's Poisson's ratio and the one selected in the TFM analysis ($d\nu=\nu_{inverse}-\nu_{forward}=0.5-\nu_{invesre}$). Black dash-curves shows the error due to only Poisson's ratio mismatch, assuming that the displacement field has been quantified perfectly. Blue, red, and yellow curves represent the corresponding error to BD= 0.05, 0.25, and 2 μm^{-2}, respectively. The error is computed for mean normal stress (left), shear stress (middle), and average of all components of stress (right). Errorbars represent standard deviation.*

The analytical solution used to draw the plots in Fig. S1 are provided in the following section. Figure S2 (which is the same as Fig. S1.a, but provides more details about the geometry of the substrate) shows the idealised case of a TFM problem, in which uniform normal and shear traction forces are being applied to the upper surface of a substrate, while the bottom surface is fixed in three coordinate directions. Here, the aim is to theoretically evaluate how a mismatch in Poisson's ratio can cause error in TFM problems. To this end, the problem shown in Fig. S2 can be considered as a Timoshenko-Ehrenfest cantilever beam. Since, unlike Euler-Bernoulli beam, the governing differential equation for such a beam has a second-order partial derivative, which enables it to take in to account the shear deformation effects, using such formulation is suitable for modelling the short beam presented in Fig. S2.

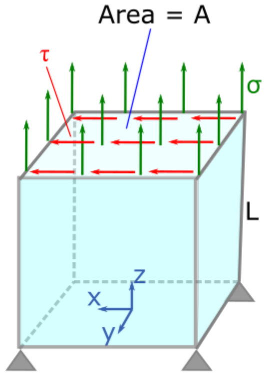

Supplementary Figure S2. *Idealised TFM problem*

For a Timoshenko beam with axial effects under static loads, the displacements are assumed to be in the following form [2]:

$$\begin{cases} u = \dfrac{-\upsilon\sigma}{E}x - z.\varphi(x) \\ v = \dfrac{-\upsilon\sigma}{E}y \\ w = \dfrac{\sigma}{E}x + \omega(X) \end{cases} \quad \text{(S1)}$$

in which (x, y, z) are the coordinates of a point of the beam and (u, v, w) are the components of displacement vector in three coordinates directions. φ shows the angle of rotation of a plane normal to neutral axis and ω is the displacement of neural axis in the z-direction [2].

The governing equations are the following system of ordinary differential equations:

$$\begin{cases} \dfrac{d^2}{dz^2}\left(EI\dfrac{d\varphi}{dz}\right) = 0 \\ \dfrac{d\omega}{dz} = \varphi - \dfrac{1}{\kappa AG}\dfrac{d}{dz}\left(EI\dfrac{d\varphi}{dz}\right) \end{cases} \quad \text{(S2)}$$

where A is the cross-section area, I is the second moment of area around y-axis, E represents the Young's modulus, G is the shear modulus and κ is the Timoshenko shear coefficient which for a rectangular cross-section is $\frac{10(1+\nu)}{12+11\nu}$, in which ν is Poisson's ratio.

Solving equations S2 in conjunction with the boundary conditions shown in Fig. S2, gives the displacement vector as below:

$$\begin{cases} u = \frac{-\upsilon\sigma}{E}x + \frac{\tau}{\kappa G}z + \frac{\tau AL}{2EI}z^2 - \frac{\tau A}{6EI}z^3 \\ v = \frac{-\upsilon\sigma}{E}y \\ w = \frac{\sigma}{E}x - \frac{\tau A}{EI}xz(L - \frac{z}{2}) \end{cases} \quad \text{(S3)}$$

Now, the displacement vector is known, and we can solve the inverse problem. In other words, we can compute reconstructed traction forces using the given displacement field. The only point is that for the inverse problem we assume that the material is incompressible i.e. $\upsilon' =$

0.5. Then, the error due to Poisson's ratio mismatch can be obtained by comparing reconstructed tractions with the applied ones (the ones shown in Fig. S2). First, we need to find infinitesimal strains as follows [3]:

$$\begin{cases} \varepsilon_{xx} = {\partial u}/{\partial x} \\ \varepsilon_{yy} = {\partial v}/{\partial y} \\ \varepsilon_{zz} = {\partial w}/{\partial z} \\ \varepsilon_{xy} = \frac{1}{2}({\partial u}/{\partial y} + {\partial v}/{\partial x}) \\ \varepsilon_{xz} = \frac{1}{2}({\partial u}/{\partial z} + {\partial w}/{\partial x}) \\ \varepsilon_{yz} = \frac{1}{2}({\partial v}/{\partial z} + {\partial w}/{\partial y}) \end{cases} \tag{S4}$$

$$\begin{cases} \varepsilon_{xx} = -\frac{\upsilon\sigma}{E} \\ \varepsilon_{yy} = -\frac{\upsilon\sigma}{E} \\ \varepsilon_{zz} = \frac{\sigma}{E} - \frac{\tau AxL}{EI} + \frac{\tau Axz}{EI}\Big|_{z=L} = \frac{\sigma}{E} \\ \varepsilon_{xy} = 0 \\ \varepsilon_{xz} = \frac{\tau(1+\nu)}{E} \\ \varepsilon_{yz} = 0 \end{cases} \tag{S5}$$

Then stresses are calculated from strains using constitutive law, which is usually written in the following form for linear elastic materials [3]:

$$\sigma_{ij} = \lambda\varepsilon_v\delta_{ij} + 2\mu\varepsilon_{ij} \tag{S6}$$

$$\lambda = \frac{E\upsilon'}{(1+\upsilon')(1-2\upsilon')} \qquad \mu = \frac{E}{2(1+\upsilon')}$$

where λ and μ are Lamé coefficients and υ' is the Poisson's ratio used in the inverse problem, which is not necessarily equal to true underlying material's Poisson's ratio and ε_v is the volumetric strain ($\varepsilon_{xx} + \varepsilon_{yy} + \varepsilon_{zz}$). A closer examination of the constitutive law reveals that this equation highlights the dependence of the shear ($i \neq j$) and normal ($i = j$) stresses on the Poisson's ratio, indicating how any mismatch in the assumed Poisson's ratio and the real underlying material Poisson's ratio will be translated into an error in both shear and normal stresses. Additionally, since the shear and normal stresses have a distinct dependence on the Poisson's ratio (the shear stress component is only a function of μ and the normal stress is a function of both μ and λ) we would expect higher errors in the normal stress compared to those in the shear stress. Specifically, the assumption of incompressibility ($\upsilon \cong 0.5$) may generate very large errors in the normal stresses. As has been shown previously, achieving sufficiently high bead densities (BD) is a key determinant of TFM accuracy, yet the intimate dependence of the stress calculation on the Poisson's ratio indicates that even with perfect quantification of the substrate displacements, the predominate source of error in TFM could result from misestimating this material property.

However, using Eq. S6 is not suitable for incompressible materials, because substituting $\upsilon' = 0.5$ in it, leads to an infinite λ. Actually, because we can apply any pressure to an incompressible material without changing its shape, the stress cannot be uniquely determined from strains using Eq. S6. To resolve this issue, since bulk modulus $K = {}^{E}\!/_{3(1-2\upsilon)}$ and mean normal stress $p = K\varepsilon_v$, we can rewrite Eq. S6 in the following form:

$$\sigma_{ij} = \frac{3\upsilon'}{1+\upsilon'} p\delta_{ij} + 2\mu\varepsilon_{ij} \xRightarrow{\upsilon'=0.5} \sigma_{ij} = p\delta_{ij} + 2\mu\varepsilon_{ij} \tag{S7}$$

Here, the mean normal stress, p, is considered as an independent variable and can usually be calculated by solving equilibrium equation together with appropriate boundary conditions. Here, it can be deduced from Fig. S2 that $p = {}^{\sigma}\!/_{3}$. It worth noting that such formulation is also applied in defining the "Hybrid element" in ABAQUS, which has been designed to model incompressible materials. Assuming a 10-by-10 square as the cross section and $\sigma = \tau$, all component of stress tensor can be determined as follows:

$$\begin{cases} \sigma_{xx} = \dfrac{\sigma(1-2\upsilon)}{3} \\ \sigma_{yy} = \dfrac{\sigma(1-2\upsilon)}{3} \\ \sigma_{zz} = \sigma \\ \sigma_{xy} = 0 \\ \sigma_{xz} = \dfrac{\sigma(1+\upsilon)}{1.5} \\ \sigma_{yz} = 0 \end{cases} \tag{S8}$$

Eq. S8 shows that the reconstructed mean normal stress is $p^{reconstructed} = \frac{\sigma(5-4\upsilon)}{9}$ and reconstructed shear stress is $\tau^{reconstructed} = \frac{\sigma(1+\upsilon)}{1.5}$. Thus, the absolute value of error due to Poisson's ratio will be:

$$Error^{in\ mean\ normal\ stress} = \frac{2(1-2\upsilon)}{3} = \frac{4\,\delta\upsilon}{3} \tag{S9}$$

$$Error^{in\ shear\ stress} = \frac{1-2\upsilon}{3} = \frac{2\,\delta\upsilon}{3}$$

in which $\delta\upsilon$ is the mismatch in Poisson's ratio. Plots displaying the variation of such errors are shown with dash-lines in Fig. S1.g. Average error is the mean of error in all six components of stress, which is computed as $Error^{average} = \delta\upsilon$ in this problem.

Supplementary Note 2- 2D behaviour

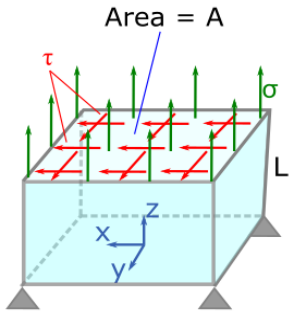

Supplementary Figure S3. *Idealised TFM problem to investigate 2D behaviour*

As can be deduced from Fig. 2b.i, for 2.5D and 3D cases, the intrinsic error due to Poisson's ratio is negligible. In other words, errors, which reflect the degree of descerpancy between the applied traction forces and the reconstructed ones, seem to be independent of the Poisson's ratio. Therefore, the error-ν curve is represented by a flat line, especially when bead density is sufficiently large (i.e. BD=2). Ideally, when the displacement field is quantified perfectly the error is zero, irrespective of the magnitude of Poisson's ratio. However, in 2D case perfect quantification of the displacement field is not possible as we discard the axial component. As a result, Fig. 2b.i shows that for 2D case, Error versus Poisson's ratio plot is an increasing curve rather than a flat line.

To investigate the mechanisms governing the observed 2D behaviour, here, we consider an idealised problem, for which an analytical solution is available. Fig. S3. shows the geometry and boundary conditions; A normal and two shear tractions are acting on the top surface, while the lower surface in constrained in all directions. This is similar to the boundary conditions applied in Fig. 2a.i to obtain plots shown in Fig. 2b.i. The only difference is that the traction forces are being applied to the whole upper face rather than a small circular region. This enables us to provide an analytical solution for the problem which helps understanding the underlying mechanisms involving in 2D case.

The displacement field can be obtained by solving equations S1 and S2 along with the boundary conditions, as presented in Note 1. However, in 2D case, only the lateral components of displacement (i.e. *u* and *v*) are recorded and the axial component (i.e. *w*) is ignored, therefore, by assuming perfect quantification of the lateral components, the recorded displacements can be written as:

$$\begin{cases} u = \dfrac{-\upsilon\sigma}{E}x + \dfrac{2\tau(1+\nu)}{\kappa E}z + \dfrac{\tau AL}{2EI}z^2 - \dfrac{\tau A}{6EI}z^3 \\ v = \dfrac{-\upsilon\sigma}{E}y + \dfrac{\tau(1+\nu)}{2\kappa E}z + \dfrac{\tau AL}{2EI}z^2 - \dfrac{\tau A}{6EI}z^3 \\ w = 0 \end{cases} \tag{S10}$$

Then, infinitesimal strains are obtained using equation S4:

$$\begin{cases} \varepsilon_{xx} = -\dfrac{\upsilon\sigma}{E} \\ \varepsilon_{yy} = -\dfrac{\upsilon\sigma}{E} \\ \varepsilon_{zz} = 0 \\ \varepsilon_{xy} = 0 \\ \varepsilon_{xz} = \dfrac{1}{2}(\dfrac{\tau}{G} + \dfrac{\tau ALz}{EI} - \dfrac{\tau Az^2}{2EI})\Big|_{@z=L} = \dfrac{1}{2}(\dfrac{2\tau(1+\nu)}{E} + \dfrac{\tau AL^2}{2EI}) \\ \varepsilon_{yz} = \dfrac{1}{2}(\dfrac{\tau}{G} + \dfrac{\tau ALz}{EI} - \dfrac{\tau Az^2}{2EI})\Big|_{@z=L} = \dfrac{1}{2}(\dfrac{2\tau(1+\nu)}{E} + \dfrac{\tau AL^2}{2EI}) \end{cases} \tag{S11}$$

For a conventional TFM experiment, the dimensions of the substrate top surface are much larger than its height, thus, $AL^2 << l$. Therefore, shear strains on z plane can be written as $\varepsilon_{xz} = \varepsilon_{yz} \cong \frac{\tau(1+\nu)}{E}$. Furthermore, to simplify the problem, again we assume $\tau=\sigma$.

Now, to obtain stress components, Eq. S11 needs to be substituted into the constitutive equation (i.e. Eq. S7). However, mean normal stress, p, is unknown and should be quantified from boundary conditions and in 2D case, a full description of them is not available, as the axial component of displacement is neglected. This is a key issue that generates error in 2D case. As a result, an accurate estimate of the mean normal stress may not be provided. Here, we simply can assume $p=K.\sigma/3$, where K is a multiplier ranging between 0 and 1. When $K=1$, the mean normal stress is obtained accurately, i.e. $p=\sigma/3$. Therefore, the stress components can be written as:

$$\begin{cases} \sigma_{xx} = \frac{\nu\sigma}{1+\nu}(K-1) \\ \sigma_{yy} = \frac{\nu\sigma}{1+\nu}(K-1) \\ \sigma_{zz} = \frac{K\nu\sigma}{1+\nu} \\ \sigma_{xy} = 0 \\ \sigma_{xz} = \tau \\ \sigma_{yz} = \tau \end{cases} \tag{S12}$$

Now, the stress components have been reconstructed, the corresponding error can be computed by comparing them with the applied ones. Using Eq. S17, the error can be written as:

$$Error = \frac{1+3\nu(1-K)}{3(1+\nu)} \tag{S13}$$

The last issue is to estimate K, which needs to be found according to problems requirement. Here, for the sake of simplicity, we assume $K=1-2\nu$. This means, when the substrate is incompressible, i.e. it undergoes no volume change under applied traction forces, $K=0$. On the other hand, when $\nu=0$, corresponding to the maximum compressibility of the substrate, $K=1$. Therefore, the theoretical error will be obtained from the following equation:

$$Error\ (\%) = \frac{1+6\nu^2}{3(1+\nu)} \times 100 \tag{S14}$$

Figure S4 compares the analytical error, from Eq. 14, and the one obtained from FEM simulations, as shown in fig. 2b.i. This demonstrates that the FEM simulations and analytical solution curves follow the same pattern. It should be noticed that the boundary conditions for FEM simulations are different from that for analytical solution, since the tractions are applied only on a small circular region for FEM simulations. Therefore, we would not expect that the FEM curves approach the analytical curve as the bead density increases.

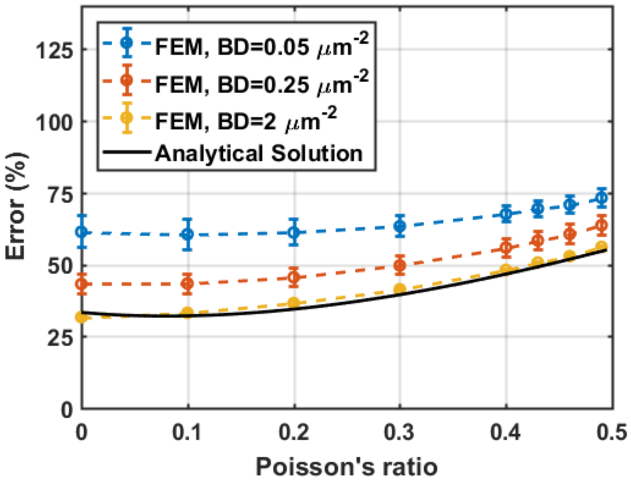

Supplementary Figure S4. *Comparison of intrinsic error obtained from finite element simulations, with bead density=0.05, 0.25, and 2 μm^{-2}, shown with blue, red, and yellow lines, respectively, with the analytical solution (Eq. S14). This shows that both FEM and analytical solutions follow the same trend.*

This study has revealed that for 2D case, ignoring the axial displacements has two major effects on reconstructing the tractions: 1) wrong estimation of axial strains and consequently normal stress (σ_{zz}) and 2) wrong estimation of mean normal stress. Due to these two effects, the intrinsic error versus Poisson's ratio curve for 2D case, unlike 2.5D and 3D cases, would not be a flat line.

Figure 2b. ii shows that for 2.5D and 3D cases, error is an increasing function of mismatch in Poisson's ratio. In other words, as expected, increasing the mismatch between the real underlying material Poisson's ratio (used in forward problem) and that used in TFM analysis (i.e. the inverse problem) leads to an increase in deviation of the reconstructed tractions from the simulated ones. However, the figure shows that 2D case does not follow the same trend; i.e. for 2d case, the error-mismatch curve is decreasing at the beginning and then it becomes increasing.

It should be noted again that for figures 2b.ii, ν for the forward problem ranges between 0 and 0.5 while for the inverse problem the Poisson's ratio is equal to 0.5. Therefore, the mismatch in the Poisson's ratio is $\delta\upsilon = 0.5 - \upsilon$. Keeping this point in mind, although the 2D behaviour may look complex at first glance, it could be justified simply; The biphasic relationship for 2D case actually originates from two sources: 1) By increasing ν in the forward problem, the Poisson's ratio mismatch, $\delta\nu$, decreases, consequently, like 2.5D and 3D cases, the pure mismatch error decreases. 2) By increasing ν in the forward problem, as shown in Fig. 2b.i, the intrinsic error increases. For 2D case, the total error would be the sum of these two sources. Therefore, as shown in Fig. S5a the error shows such a biphasic behaviour. It is worth noting that for 2.5D and 3D cases, as shown in Fig.1b.ii, the intrinsic error (source 1) is negligible. Therefore, the pure mismatch error would be the only source of error for such cases, implying a direct relationship between error and $\delta\nu$. In other word, the total error would be sum of mismatch error and intrinsic error. The latter one, is negligible for 2.5D and 3D cases while for 2D case it cannot be ignored.

To investigate the mechanisms governing the observed 2D behaviour more precisely, we again consider the idealised problem shown in Fig.S3. The boundary conditions are similar to

those applied in Fig. 2a.i to obtain plots shown in Fig. 2b.ii. The only difference is that the traction forces are being applied to the whole upper face rather than a small circular region.

As shown above, the displacement field and strains can be obtained using Eq. S10 and Eq. S11, respectively. Again, to simplify the problem, we assume $\tau=\sigma$ and the shear strains on *z* plane can be written as $\varepsilon_{xz} = \varepsilon_{yz} \cong \frac{\tau(1+\upsilon)}{E}$. Similar to the previous case, the mean normal stress is assumed *p=K.* σ*/3* where the multiplier is again *K=1-2*ν.

To compute the mismatch error, unlike the intrinsic error, the Poisson's ratio in inverse problem (ν'=0.5) would not the same as that in the forward problem (ν). Now, by substituting strains into the constitutive equation, the stress components would be computed as:

$$\begin{cases} \sigma_{xx} = \frac{\sigma}{3}(K - 2\upsilon) \\ \sigma_{yy} = \frac{\sigma}{3}(K - 2\upsilon) \\ \sigma_{zz} = \frac{K\sigma}{3} \\ \sigma_{xy} = 0 \\ \sigma_{xz} = \sigma\frac{1+\upsilon}{1.5} \\ \sigma_{yz} = \sigma\frac{1+\upsilon}{1.5} \end{cases} \tag{S15}$$

Now, the stress components have been reconstructed, the corresponding error can be computed by comparing them with the applied ones. Thus, the error can be written as:

$$Error(\%) = \frac{2}{9}(|1 - 4\upsilon| + 2 - \upsilon) \times 100 \tag{S16}$$

Figure S5b compares the analytical error, from Eq. S16, and the one obtained from FEM simulations, as shown in fig. 2b.i. This demonstrates that the FEM simulations and analytical solution curves represent similar behaviour. Again, it should be noticed that the boundary conditions for FEM simulations are different from that for analytical solution, since the tractions are applied only on a small circular region for FEM simulations. Therefore, we would not expect that the FEM curves approach the analytical curve as the bead density increases.

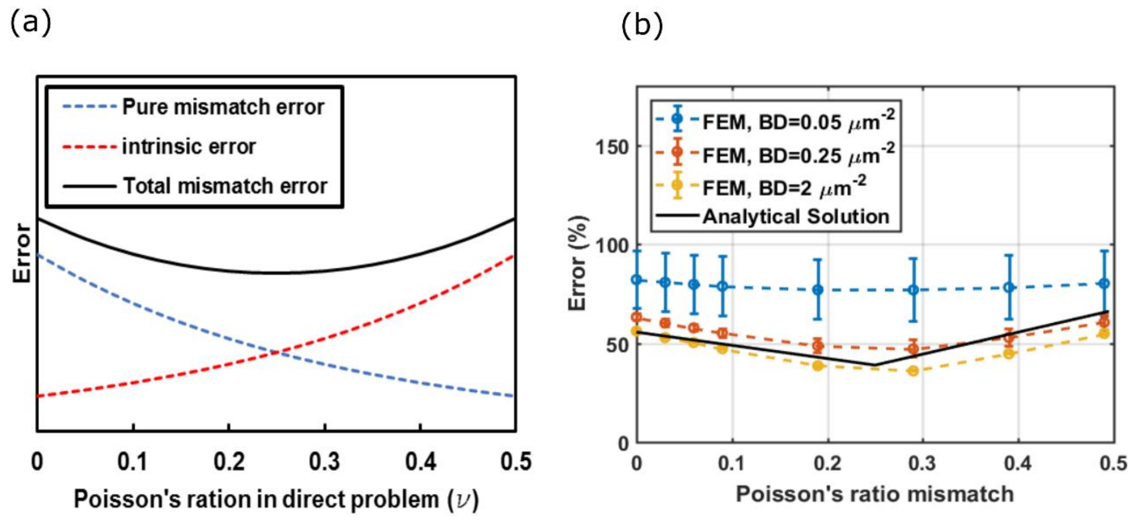

Supplementary Figure S5. (a) *Schematic plot representing the two sources of error for 2D case in computing mismatch error: (1) Pure mismatch error: for a smaller mismatch between real underlying material Poisson's ratio (ν) and the ratio used for TFM analysis (i.e. 0.5), a smaller pure mismatch error would be expected (2) Intrinsic error which directly depends on ν. Total mismatch error would be sum of intrinsic and pure mismatch error.* ***(b)*** *Comparison of total mismatch error obtained from finite element simulations, with bead density=0.05, 0.25, and 2 μm^{-2}, shown with blue, red, and yellow lines, respectively, with the analytical solution (Eq. S16). Analytical solution curve shows a biphasic relationship as well as FEM curves. This shows that both FEM and analytical solutions follow the same trend. Errorbars represent standard deviation.*

Similar to the intrinsic error, ignoring the axial displacement leads to wrong estimation of axial strain and mean normal stresses. As a result, the magnitude of error would not be zero when the displacement field is perfectly quantified. Additionally, unlike 2.5D and 3D cases, the error curve for 2D case follows a biphasic relationship.

Supplementary Note 3- Illustration of traction force distribution

In this section the distribution of normal and shear traction forces obtained from solving forward and inverse problem is presented. Additionally, the distribution of error is shown by comparing the simulated and reconstructed traction forces. Figures S6 and S7 correspond to investigation of intrinsic and mismatch error, respectively. The distribution of shear and normal traction forces in addition to errors are illustrated for 2D, 2.5D, and 3D cases. Figures S8 shows the impact of traction size on the error in estimating traction forces, the corresponding stress and error plots are also depicted for 2D and 2.5D cases in the figure.

It should be noted that the error is computed over the traction zone using the following equation:

$$Error(\%) = \frac{\sum_{j=i}^{3}\sum_{i=1}^{3}\sum_{node=1}^{n}\left|\sigma_{ij,node}^{invesre} - \sigma_{ij,node}^{forward}\right|}{\sum_{j=i}^{3}\sum_{i=1}^{3}\sum_{node=1}^{n}\left(\left|\sigma_{ij,node}^{forward}\right|\right)} * 100 \quad \text{(S17)}$$

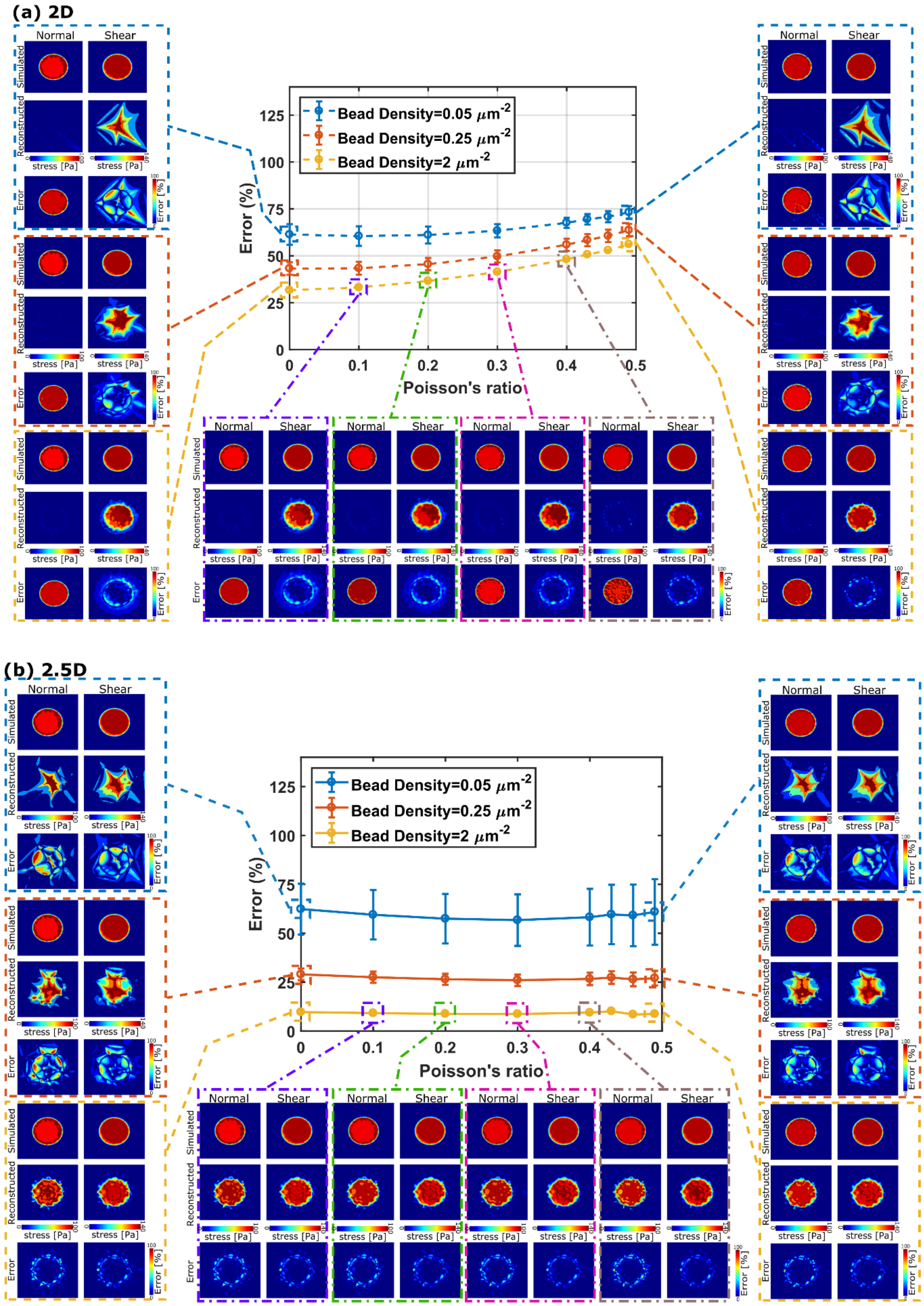

(a) 2D
Normal
Shear
Simulated
Reconstructed
Error
stress [Pa]
Error [%]
Bead Density=0.05 μm-2
Bead Density=0.25 μm-2
Bead Density=2 μm-2
Error (%)
Poisson's ratio
0
25
50
75
100
125
0.1
0.2
0.3
0.4
0.5
(b) 2.5D

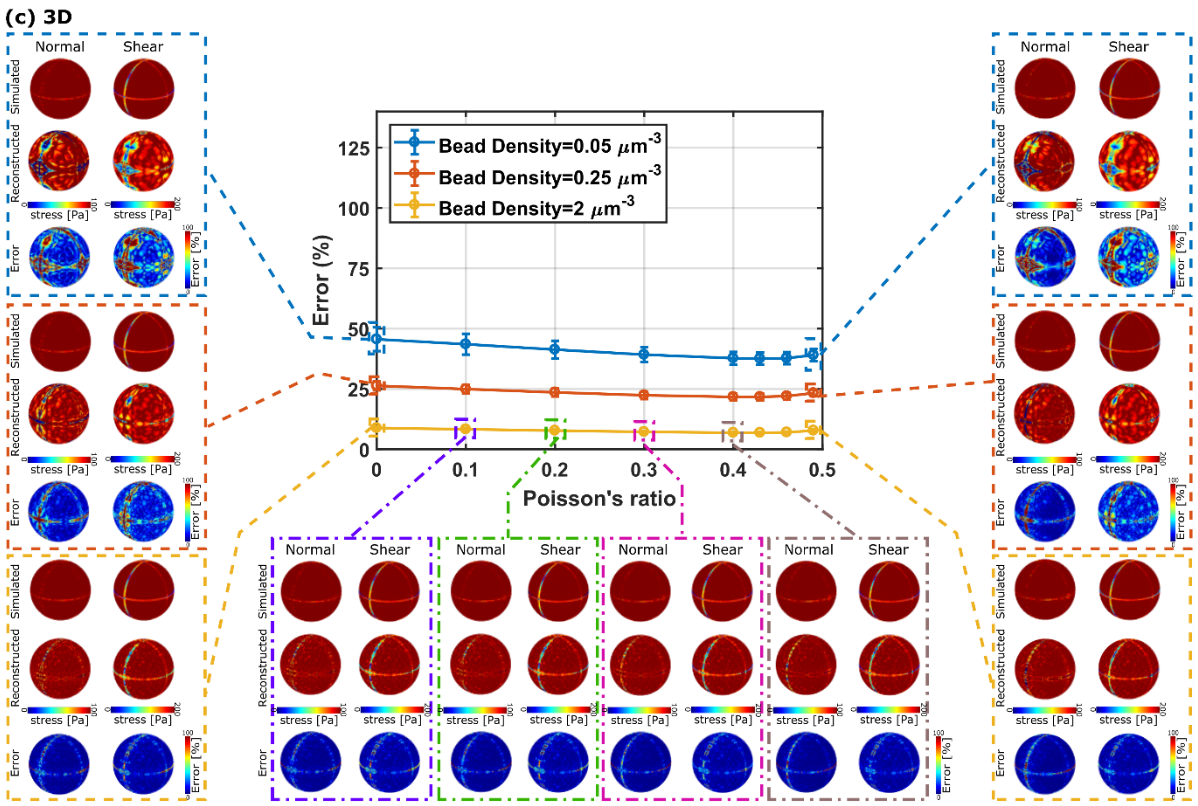

Supplementary Figure S6. *Illustration of normal and shear traction force distributions and corresponding errors in investigating the impact of intrinsic error for* ***(a)*** *2D* ***(b)*** *2.5 D and* ***(c)*** *3D cases. Graphs in the centre show the magnitude of error. Plots on left and right show the impact of bead density on distribution of traction forces and corresponding errors when the Poisson's ratio is 0 and 0.49, respectively, while plots on the bottom shows the effect on Poisson's ratio on the distribution of normal and shear tractions and corresponding errors. Errorbars represent standard deviation.*

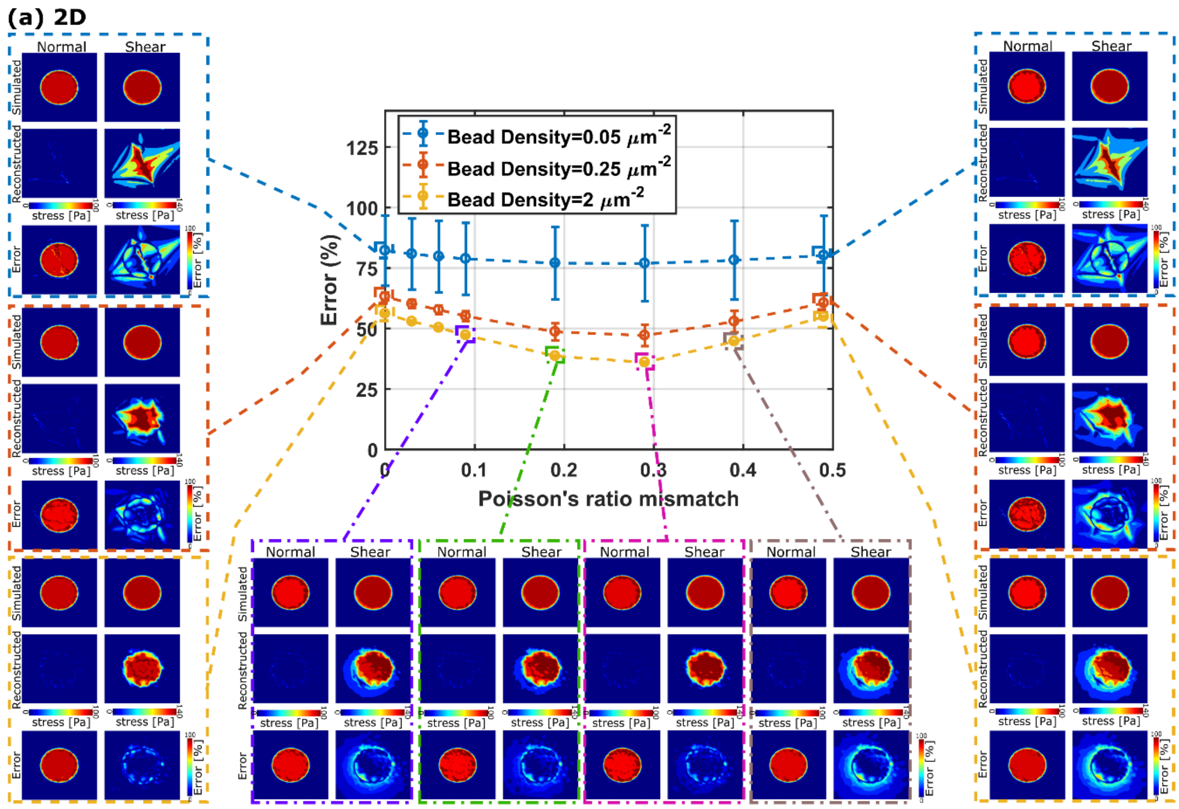

(b) 2.5D

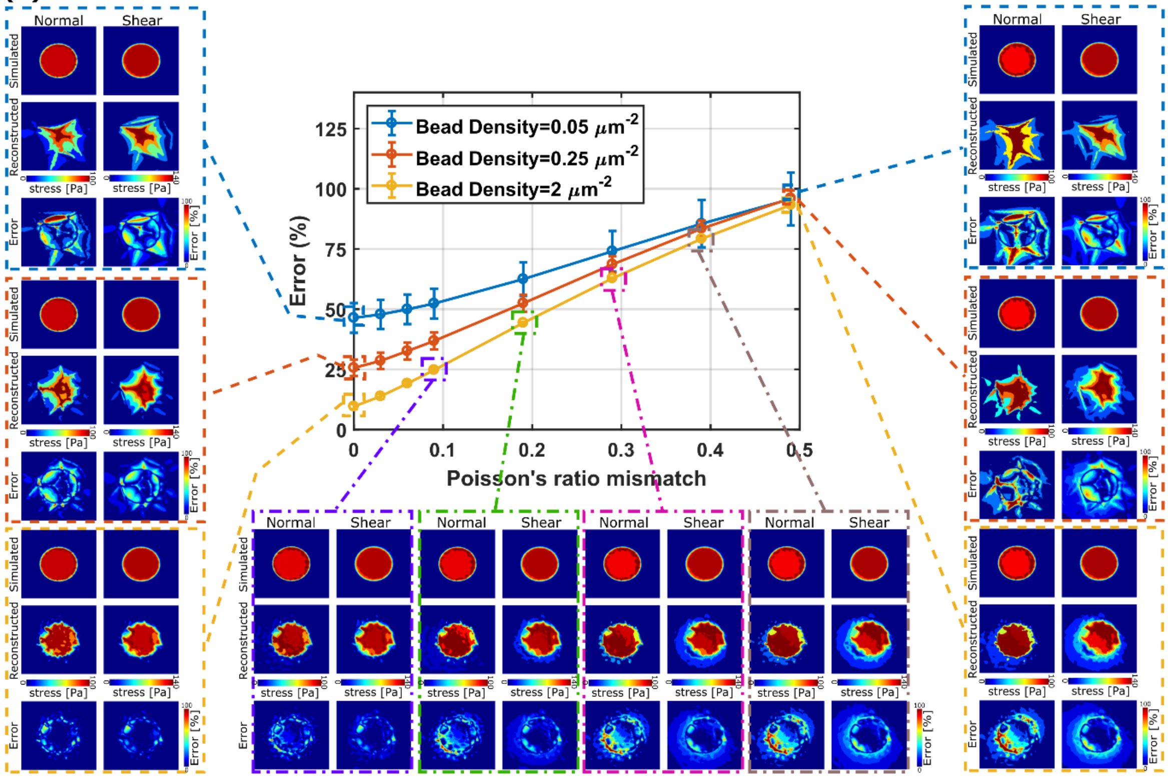

(b) 3D

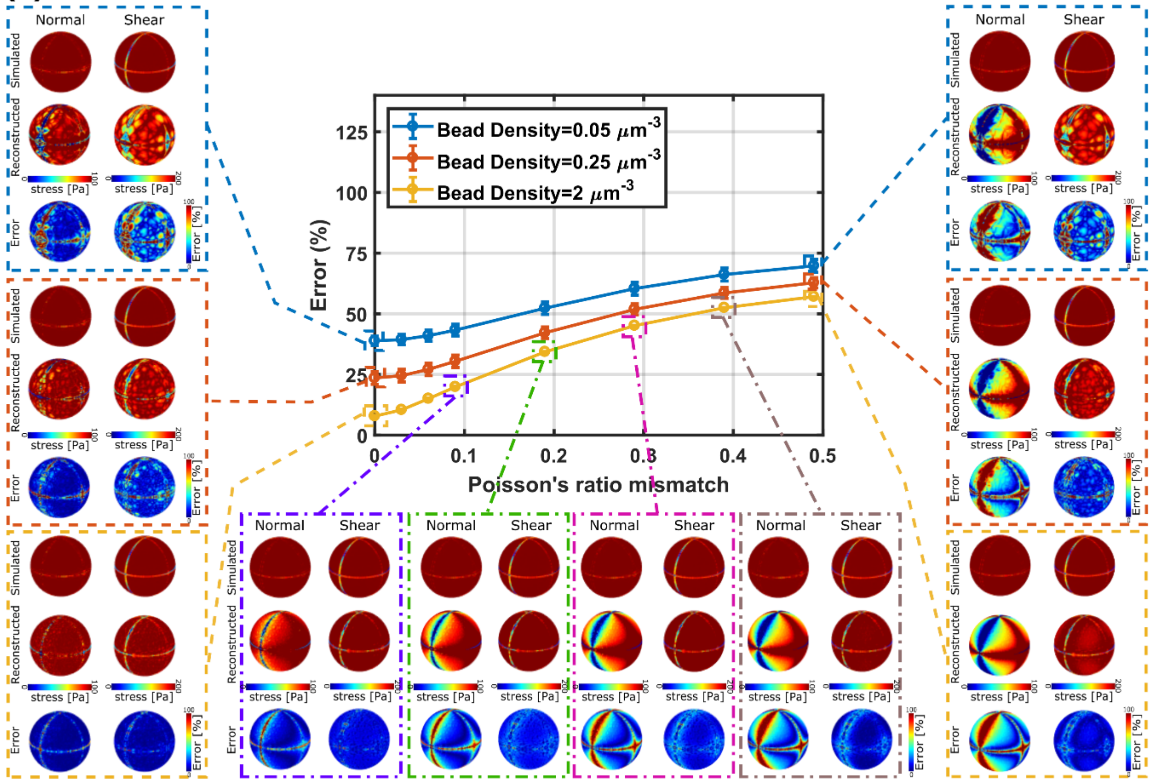

Supplementary Figure S7. *Illustration of normal and shear traction force distributions and corresponding errors in investigating the impact of mismatch error for* ***(a)*** *2D* ***(b)*** *2.5 D and* ***(c)*** *3D cases. Graphs in the centre show the magnitude of error. Plots on left and right show the impact of bead density on distribution of traction forces and corresponding errors when the Poisson's ratio mismatch is 0 and 0.49, respectively, while plots on the bottom shows the effect on Poisson's ratio mismatch on the distribution of normal and shear tractions and corresponding errors. (*$\nu_{forward}$ *= 0, 0.1, 0.2, 0.3, 0.4, 0.5;* $\nu_{inverse}$ *= 0.5). Errorbars represent standard deviation.*

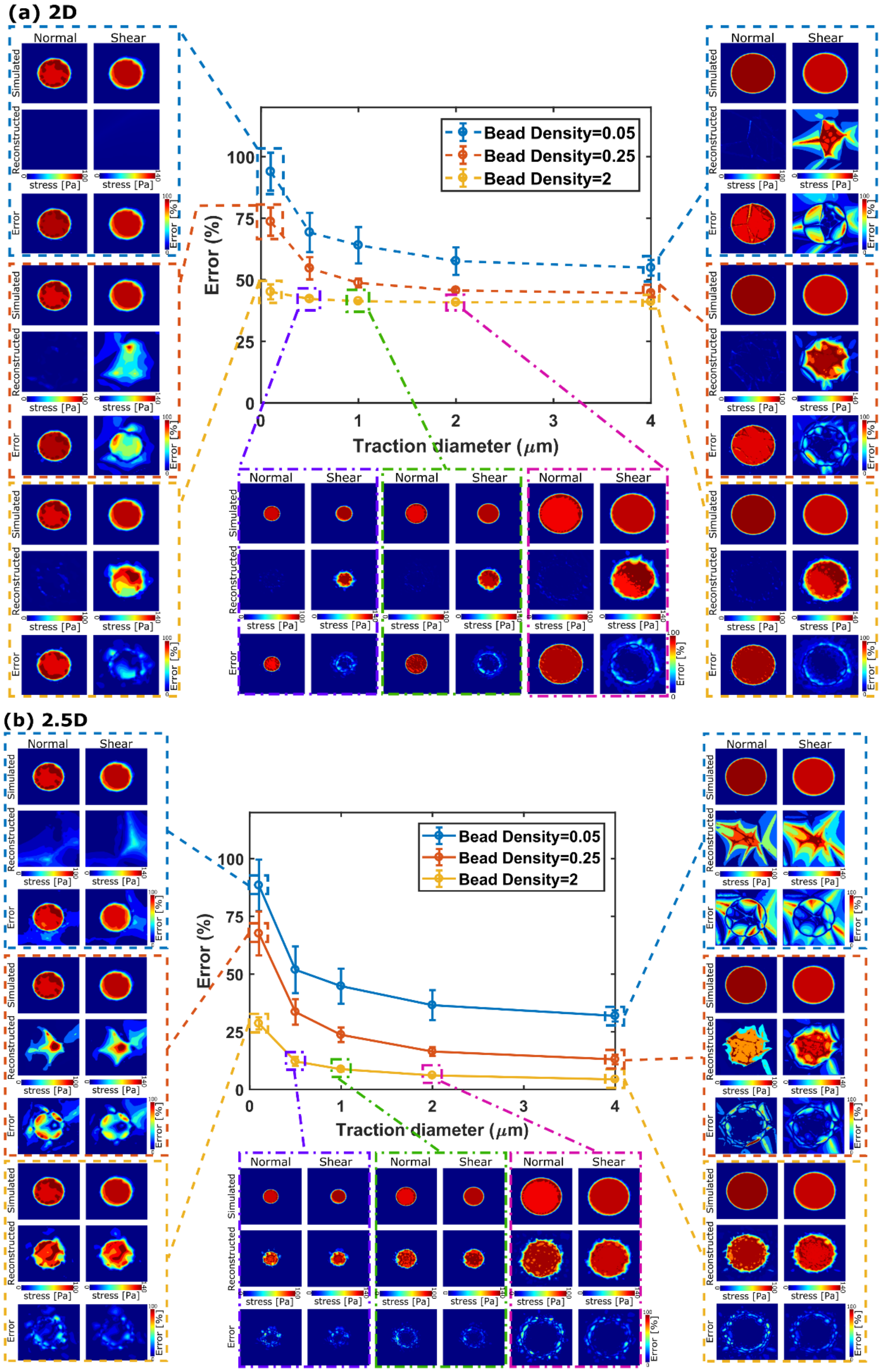

Supplementary Figure S8. *Illustration of normal and shear traction force distributions and corresponding errors in investigating the impact of traction size for* ***(a)*** *2D* ***(b)*** *2.5 D. Graphs in the centre show the magnitude of error. Plots on sides show the impact of bead density, while plots on the bottom shows the effect of traction diameter on the distribution of normal and shear tractions and corresponding errors. Errorbars represent standard deviation.*

The normal and shear stresses were depicted only on a small region influenced by the cell in **Fig. 3b**. To evaluate noise levels at regions located far away from the cell during the RBL activation experiment, the tractions are shown on a larger area in **Fig. S9**. Negligible noises far away from the cell supports the accuracy of displacement acquirement and traction reconstruction algorithms employed in this study.

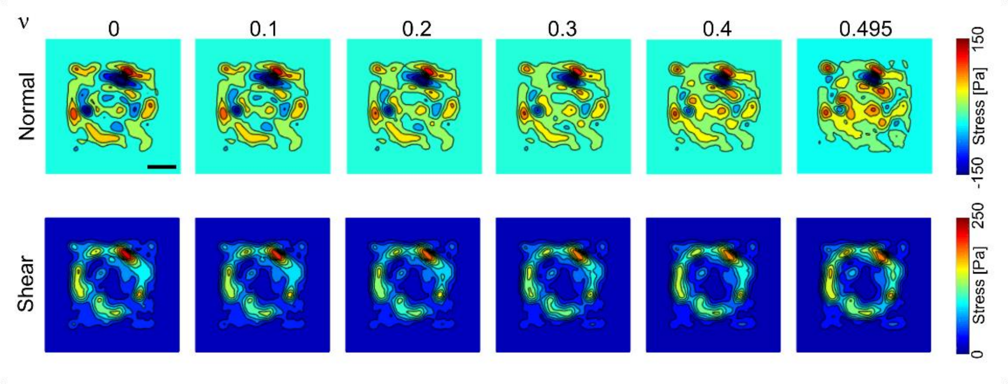

Supplementary Figure S9. *Magnitude of the reconstructed normal and shear stresses on a larger region assuming the possible Poisson's ratio of the substrate ranges between 0-0.5. Scale = 10 μm.*

Supplementary Note 4– Sensitivity of TFM analysis to axial component of displacement

As mentioned before, in some early TFM studies, only two lateral component of displacements recorded (referred to as 2D case); However, in later investigations, quantification of normal traction forces, in addition to shear tractions, was enabled by measuring all three components of displacement (two lateral and one axial components). Such studies revealed that for some cases, the magnitude of normal stresses might not be insignificant compared to the magnitude of shear tractions. Here, we aim to derive a threshold, above which the normal tractions cannot be ignored.

Figure S10.a show the error in estimation of traction forces as a function of true underlying material Poisson's ratio for different ratios of normal (σ) to shear (τ) tractions, ranging from 0 to 1. The ratio of 0 corresponds to the condition where only shear tractions are applied to the surface. The results from the 2.5D case is shown by solid lines, for which the three displacement components are used to reconstruct the tractions, while dash lines represent 2D case, for which only lateral component of displacement are considered and the axial component is ignored. Therefore, for a given ratio of σ/τ, the difference between 2D and 2.5D curve could be attributed to ignoring the axial displacement. In other words, for a given σ/τ, when the 2D and 2.5D curves are close enough, the impact of ignoring the axial displacement would be negligible.

In order to compare the affinity of the 2D and 2.5D curves, an unpaired two-sided student's t-test was conducted to find p-value at each Poisson's ratio. Figure S10.b shows the results for different ratios of σ/τ. As can be concluded from figure S10.a, for σ/τ=0 (green lines), the difference between 2D and 2.5D curves decreases as the Poisson's ratio increases, which corresponds to an increase in p-values. For other ratios of σ/τ, the 2D and 2.5D curves converge as Poisson's ratio rises from 0 to $\sim$ 0.3 and the difference between them increases afterwards. Consequently, corresponding p-value curves show a peak at ν=$\sim$ 0.3. The region hatched with blue lines represents p-values lower than a significance level of 95%, therefore, in this region, the difference between 2D and 2.5D curves would be significant and axial displacement could not be ignored. To simplify the results more, geometric average of p-values was calculated for each ratio of σ/τ and shown in Fig. S10.c. The figure shows that when σ/τ<0.1, p-value would be greater than 5% (for significance level of 95%) and thus, the difference between 2D and 2.5D curves and consequently the error due to ignoring axial displacements would not be significant.

The threshold introduced above is expressed in terms of normal to shear traction forces ratio (σ/t=0.1). However, it might be simpler for experimentalists to work with displacements rather than traction forces, as the magnitude of displacements can be readily quantified by tracking the fluorescent beads, while finding the unknown tractions require one more computational step. Figure S10.d can be used to convert the stress ratio to displacement ratio for different Poisson's ratios. As can be deduced from the figure, the stress ratio of σ/t=0.1 is equivalent to the axial to radial displacement ratio of 0.06 to 0.1, when the Poisson's ratio is ranging from 0.5 to 0.0. Additionally, Fig. S10.d can be considered from another perspective; All lines are located above the line y=x, i.e. for all values of Poisson's ratio, $\sigma/\tau > U_{axial}/U_{lateral}$. Therefore, if the recorded axial and lateral displacement at a specific point are equal ($U_{axial}/U_{lateral}=1$), the normal stress will be greater than the shear stress ($\sigma > \tau$). In other words, reconstructed

stresses are more sensitive to the axial displacement rather than lateral components and hence, it needs to be quantified more accurately.

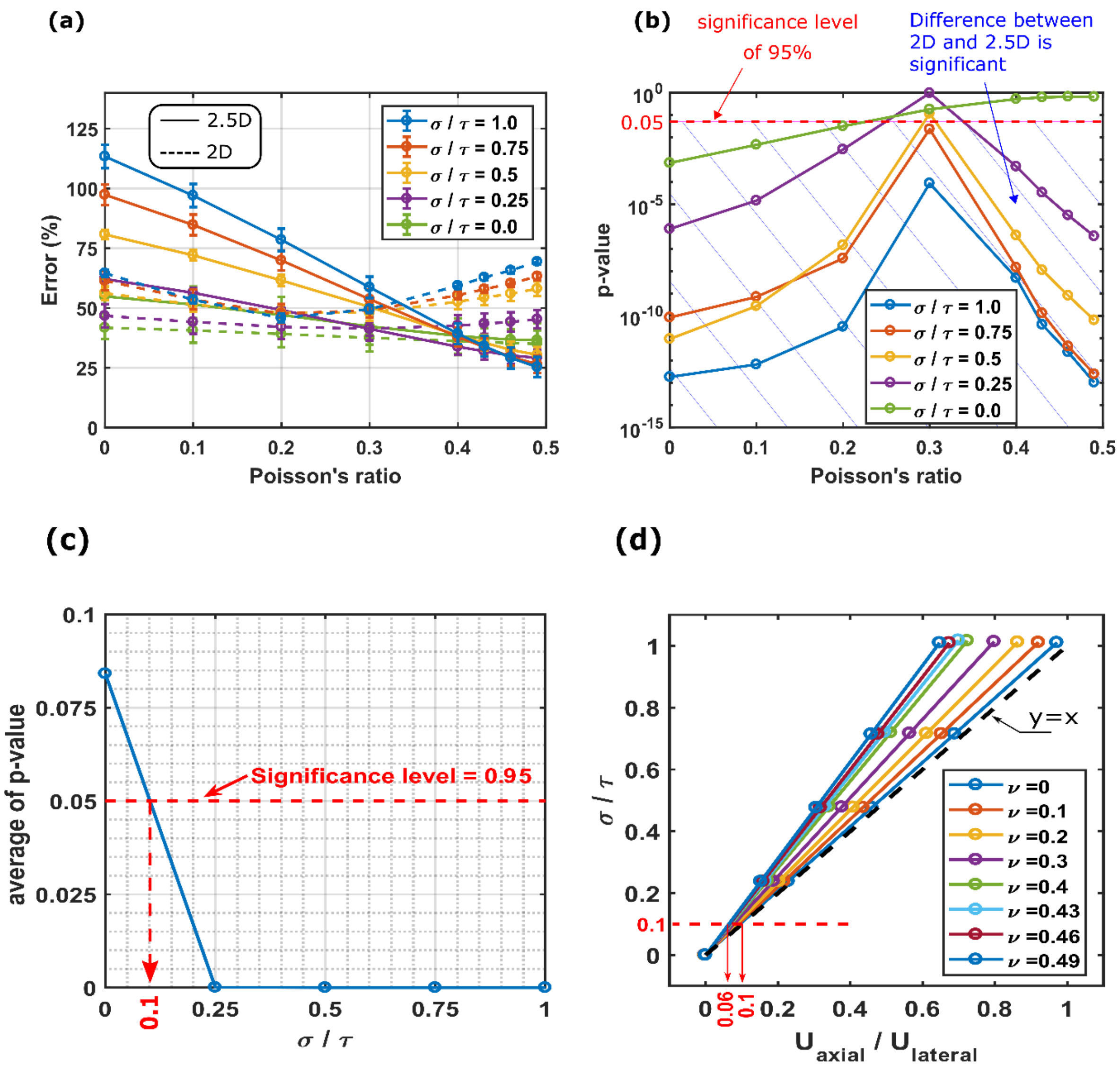

Supplementary Figure S10. *Assessing the significance of axial displacement through comparing 2D and 2.5D analyses.* ***(a)*** *Error in estimation of traction forces versus the true underlying material Poisson's ratio for different ratios of normal (σ) to shear (τ) tractions. 2.5D and 2D results are shown by solid and dash lines, respectively. Errorbars represent standard deviation.* ***(b)*** *P-values versus Poisson's ratio for different ratios of normal to shear traction forces, obtained by comparing 2D and 2.5D curves using a paired t-test.* ***(c)*** *Geometric average of p-value for each ratio of normal to shear stress. For a significant level of 95%, when σ/τ is less than 0.1, p-value would be greater than 5%, and the difference would not be significant, therefore the difference between 2D and 2.5D curves, which stems from ignoring the axial displacement, would be insignificant. In other word, neglecting axial displacement would not generate a significant error when σ/τ< 0.1.* ***(d)*** *Corresponding values of axial / lateral displacements for a range of Poisson's ratios.*

Supplementary Note 5- Green's functions vs FEM

As the aim of TFM is to quantify cell generated traction forces, one important step of TFM is to derive tractions from measured displacement field. To this end, Green's functions method (GFM) provides a simple analytical solution for the TFM problem. Green Functions have been employed to calculate the stress field from the measured displacement field analytically. These functions can be decoupled in the lateral and vertical directions when the material is incompressible[4]. However, numerical solutions such as the finite element method (FEM) provide a straightforward and robust solution to both forward and inverse problems. Furthermore, FEM benefits from some important advantages including:

- **The flexibility in simulating complicated geometries**: FEM can simulate all the relevant geometries (2D, 2.5D, 3D) and is not restricted to half space or planar geometries.
- **The ability to model materials with different degrees of compressibility**: Using FEM, one can readily apply a different Poisson's ratio and investigate its effect on the reconstructed tractions (FE frameworks is not limited to incompressible materials).
- **The ability to take the non-linearity due to large deformation into account:** FEM is not limited to small strain/small deformation problems.
- **The robustness and strength in solving boundary value problems (BVPs)** as convergency is not usually an issue.
- **The ability to model complex constitutive laws** (Non-linear, visco-elastic, poro-elastic, hyper- and hypo-elastic etc).

Therefore, for all the figures presented in the main text, we have used FEM to analyse both direct and inverse problems.

On the current note, to compare the efficiency of GFM and FEM, we solve the same problem using GFM and FEM, then compare the results obtained from both forward and inverse problems. To this end, we consider a simple TFM problem where a planar circular load is applied to the top face of a cubic substrate (See Fig. S11.a)

Suppose a tangential force is applied to top face of a half-space incompressible substrate, with linear elastic behaviour. In practice, the gel is of finite thickness and is chemically attached to a coverslip such that it can be imaged from below. For the half-space assumption to hold, the gel must be sufficiently thick such that any forces applied to the upper surface of the gel are not influenced by the fixed lower surface at the coverslip. In such case, at each point with coordinate $X = [x_1,\ x_2]^T$, located at the upper surface of the substrate, displacement vector $u = [u_1, u_2]^T$ can be obtained as follows:

$$u(X) = (G * F)(X) \tag{S18}$$

Equivalently, Eq. S18 can be written in the following form:

$$u_i(x_1, x_2) = \iint_{\Omega} \sum_{j=1}^{2} G_{ij}(x_1 - x'_1, x_2 - x'_2).\, f_j(x'_1, x'_2).\, dx'_1\, dx'_2 \tag{S19}$$

where $F = [f_1,\ f_2]^T$ shows the traction force functions, $G = \begin{bmatrix} G_{11} & G_{12} \\ G_{21} & G_{22} \end{bmatrix}$ is the matrix of Green's functions, and $*$ denoted the convolution operator. For the abovementioned TFM problem, the Green's functions are as follows:

$$\begin{cases} G_{11}(x_1, x_2) = \dfrac{1+\vartheta}{\pi E} \dfrac{1}{|X|^3}((1-\vartheta|X|^2 + \vartheta x_1^2) \\ G_{12}(x_1, x_2) = \dfrac{1+\vartheta}{\pi E} \dfrac{1}{|X|^3}\vartheta x_1 x_2 \\ G_{21}(x_1, x_2) = \dfrac{1+\vartheta}{\pi E} \dfrac{1}{|X|^3}\vartheta x_1 x_2 \\ G_{22}(x_1, x_2) = \dfrac{1+\vartheta}{\pi E} \dfrac{1}{|X|^3}((1-\vartheta|X|^2 + \vartheta x_2^2) \end{cases} \quad \text{(S20)}$$

in which $|X| = \sqrt{x_1^2 + x_2^2}$ .To evaluate the integrals in Eq. S19, discrete Fourier functions provide an efficient method. Using, convolution theorem, the Fourier transform of the convolution of G and F, is the pointwise product of their Fourier transforms:

$$\hat{u}(K) = \hat{G}(K)\, \hat{F}(K) \quad \text{(S21)}$$

where $\hat{u}$, $\hat{G}$, and $\hat{F}$ are the Fourier transforms of u, G, and F, respectively and K is the spatial wave vector.

$$\begin{cases} \hat{G}_{11}(k_1, k_2) = \dfrac{2(1+\vartheta)}{E} \dfrac{1}{|K|^3}((1-\vartheta|K|^2 + \vartheta k_2^2) \\ \hat{G}_{12}(k_1, k_2) = -\dfrac{2(1+\vartheta)}{E} \dfrac{1}{|K|^3}\vartheta k_1 k_2 \\ \hat{G}_{21}(k_1, k_2) = \dfrac{2(1+\vartheta)}{E} \dfrac{1}{|K|^3}\vartheta k_1 k_2 \\ \hat{G}_{22}(k_1, k_2) = \dfrac{2(1+\vartheta)}{E} \dfrac{1}{|K|^3}((1-\vartheta|K|^2 + \vartheta k_1^2) \end{cases} \quad \text{(S22)}$$

where $|K| = \sqrt{k_1^2 + k_2^2}$. Finally, u is obtained by computing inverse Fourier transform of $\hat{u}$.

In summary, to solve the forward problem, i.e. finding displacements from tractions, the following steps need to be carried out:

1- Consider a mesh containing *n*-by-*n* squares (i.e. *n+1* nodes in each direction). The origin could be at the centre of the grids, thus, to avoid singularity at the origin, *n* should be odd.
2- Compute G at all nodes of the grid using Eq. S20. F should also be known everywhere.
3- Compute fast Fourier transform of G and F, i.e. $\hat{G}$ and $\hat{F}$, respectively, at all nodes of an extended *2n+1*-by-*2n+1* grid.
4- Compute fast Fourier transform of u, i.e. $\hat{u}$, using equation on the extended grid.
5- Compute inverse of $\hat{u}$ and extract the proper submatrix from it to obtain u.

Unlike the forward problem, solving the inverse problem, i.e. finding the traction forces from measured displacement, is not that straightforward. Actually, the inverse problem is an ill-pose and inversing Eq. S21 may lead to highly divergent traction field. However, such a problem can be resolved using Tikhonov regularization. The aim is to minimise the following function:

$$Error(\hat{F}_\lambda) = \left\{ \left\| \hat{G}\hat{F}_\lambda - \hat{u} \right\|^2 + \lambda^2 \left\| \hat{F}_\lambda \right\|^2 \right\} \quad \text{(S23)}$$

in which λ is regularisation parameter. To obtain the minimum error, the equation $\partial Error/\partial \hat{F}_\lambda = 0$ needs to be solved. This leads to the following equation:

$$\hat{F}_\lambda = \left(\hat{G}^T\hat{G} + \lambda^2 I\right)\hat{G}^T\hat{u} \quad \text{(S24)}$$

The regularisation parameter, λ, is also determined by plotting solution norm ($\|\hat{F}_\lambda\|$) versus residual norm $\|\hat{G}\hat{F}_\lambda - \hat{u}\|$ for a range of regularisation parameters and find λ value corresponding to the cusp point in the plot.

In summary, the step to solve the inverse problem are as follows:

1- Consider a mesh containing *n*-by-*n* squares (i.e. *n+1* nodes in each direction). The origin could be at the centre of the grids, thus, to avoid singularity at the origin, *n* should be odd.
2- Compute G at all nodes of the grid using Eq. S20. u should also be known everywhere.
3- Compute fast Fourier transform of G and u, i.e. $\hat{G}$ and $\hat{u}$, respectively, at all nodes of an extended *2n+1*-by-*2n+1* grid.
4- At each node of the extended grid find $\hat{F}_\lambda$ using Eq. S24.
5- Plot residual norm $\|\hat{G}\hat{F}_\lambda - \hat{u}\|$ versus $\|\hat{F}_\lambda\|$ and find λ_{opt} corresponding to the cusp point.
6- Find $\hat{F}_\lambda$ in Eq. S24 using λ_{opt} as input.

Here, to compare the performance of GFM with that of FEM in solving TFM forward and reverse problems, we solve the following problem with both method and compare the results:

A tangential traction force is applied to a circular region on upper surface of a substrate (Fig. S11.a). To evaluate the accuracy of GFM and FEM in solving forward problem, displacements are quantified on the upper face using both methods. Fig. S11.b shows the results. As can be deduced, the displacements obtained from the two methods are very similar and the difference between the displacement vectors obtained at the all upper surface nodes is less than 0.5 %. This illustrates that for the forward problem, both FEM and GFM lead to the same results.

To evaluate the accuracy of the two methods in solving the inverse problem, the displacements obtained from the previous step, are employed to reconstruct the traction forces. Fig. S11.c shows that the reconstructed traction forces obtained from FEM and GFM are quite similar and the different between the reconstructed traction forces computed at all upper surface nodes is less than 9%.

Comparing reconstructed traction force with the applied one reveals that the error of FEM is ~ 0.5% for the inverse problem, while this error for GFM rises to less than 9%. Such comparison demonstrates that using FEM is not only useful in simulating complex geometries and material behaviour but also it benefits from a high degree of accuracy in solving TFM problems.

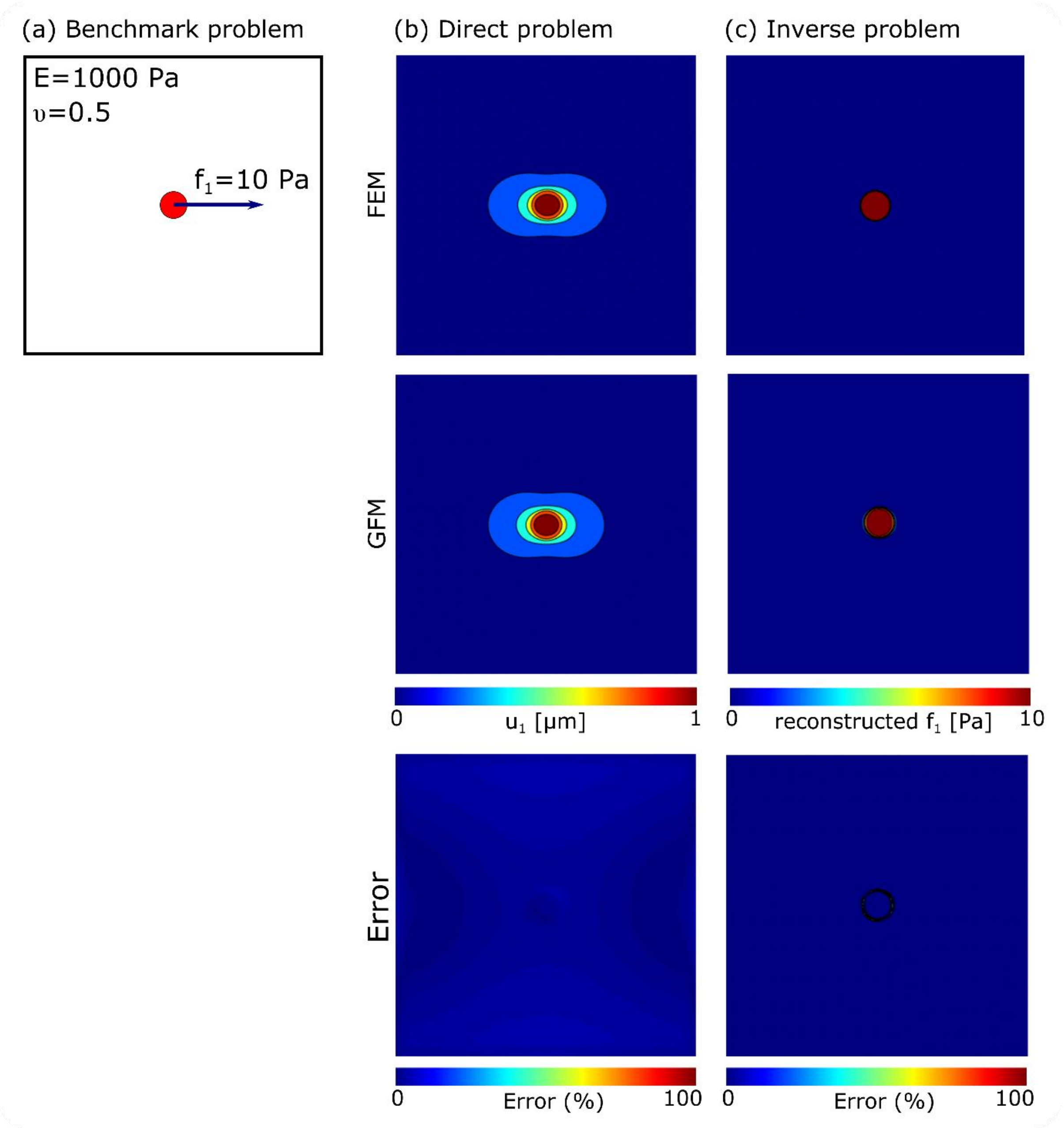

Supplementary Figure S11. *comparison of GFM with FEM.* ***(a)*** *Geometry and boundary conditions of a simple TFM problem. A shear traction was applied to a circular region on top face of a half space substrate.* ***(b)*** *The forward problem (finding displacement from the applied traction) was solved using Green's functions method (Upper) and finite element method (Middle). The results were compared and difference between the displacement fields obtained from the two methods are shown in the Lower figure.* ***(c)*** *The displacements obtained in part b were applied to the domain and tractions were reconstructed by solving the inverse problem using GFM (Upper) and FEM (Middle). The lower figure shows the difference in shear tractions obtained from GFM and FEM. This figure shows that the differences in the forward and inverse problems are less than 0.5% and 9%, respectively.*

It should be noted that FEM uses the same formulation for solving both "direct" and "inverse" problems (more details could be found in supplementary note of ref.[5]). However, as mentioned above, GFM uses different algorithms to solve such problems. Therefore, we evaluate the results of "forward" and "inverse" problems separately as follows:

For the forward problem, GFM provides exact solution considering linear elasticity and small deformations in the TFM problem. Therefore, for a pure linear elastic material, only two sources of error are available: a) Error due to discretisation of the integral, as *discrete*

convolution theorem and *discrete* Fast Fourier Transform is used to calculate the double integral on the domain (Eq. S19). b) Error due to small strain assumptions which can be negligible in GFM only when rather small tractions are applied to the domain. On the other hand, when FEM is used for the direct problem, discretisation error (which are similar to the first source of error in GFM) still exists (as tiny elements are used to discretise the domain) but the large deformation effects could be resolved using "non-linear geometry" option in FEM software (more details on how ABAQUS considers the effect of large deformation can be found in[5]). In conclusion, discretisation error exists for both methods (and is insignificant for both methods when mesh size is small) and large deformation error does not exist or is negligible for FEM and GFM. Thus, one can expect to obtain similar results using FEM and GFM. **Fig. S11b** shows that the difference between the predictions of these methods is less than 0.5 %.

For the inverse Problem, GFM does not provide the exact solution due to unstable nature of the problem. In fact, to find the unknown tractions from the known displacements, GFM solves an optimisation problem. In other words, the method minimises an error function, which may vary based on the employed regularisation scheme. Here, to solve the benchmark problem in **Fig. S11**, we used Tikhonov regularisation. In this case, two points must be considered: a) magnitude of the error is a function of regularisation parameter (λ_{opt}), which is not straightforward to obtain and may not be calculated accurately. b) The minimum value of the error function is not zero (meaning that there are still some errors in the reconstructed tractions and the solution is not exact). On the other hand, FEM treats both direct and inverse problems in the same manner, and correct application of boundary conditions converts both forward and inverse cases to a well-posed problem (See[5] and[6]). The major source of error in the FEM will be the discretisation error as mentioned above. In summary, considering the errors which encounters evaluating regularisation parameter, and bearing in mind that for inverse problem, GFM does not provide an exact solution, one may conclude that when the mesh size is sufficiently small, the recovered tractions using FEM are slightly more reliable than those obtained from GFM as supported in the results of **Fig. S11c** (~0.5% error for FEM versus ~9% error for GFM). Other studies have also shown that GFM solutions for the inverse problem are not exact and the error is a function of regularization parameter[7–9]. Additionally, it has been shown that for some specific inverse problems (convection-dominated convection–diffusion problems), in comparison to FEM, Tikhonov regularisation leads to less accurate discrete solutions[10]. It should be noted that the difference between results of FEM and GFM is not substantial (less than 9% **Fig. S11**).

Supplementary Note 6- Noise induced uncertainties in tractions

An important point that should be addressed regarding **Fig. 3** is to evaluate the effect of displacement noise. Since uncertainties are involved in determining the position of beads, and consequently in the displacement field, the computed tractions are not also accurate. To determine the localisation uncertainty of the method used in **Fig. 3**, we calculated the

ensemble standard deviation in the displacement of a subset of beads (unperturbed by any cellular force generation and corrected for 3D drift, as suggested by Plotnikov, Sergey V., et al.[1]) relative to their initial position over 100 time points (100 s). For an area with minimal background intensity the localisation uncertainty was approximately 4 nm in the lateral direction and 7 nm in the vertical direction (**Fig. S12a**). Then, a FEM model was developed in ABAQUS and a set of displacements was applied to the nodes on top face of the geometry. The exerted displacement followed a normal distribution with the same standard deviations as those shown in **Fig. S12a**. After running the model, stresses at the top nodes were extracted from the software and the results were shown in **Fig.S12b**. The standard deviation is approximately 7 Pa and 17 Pa for shear and normal stresses, respectively.

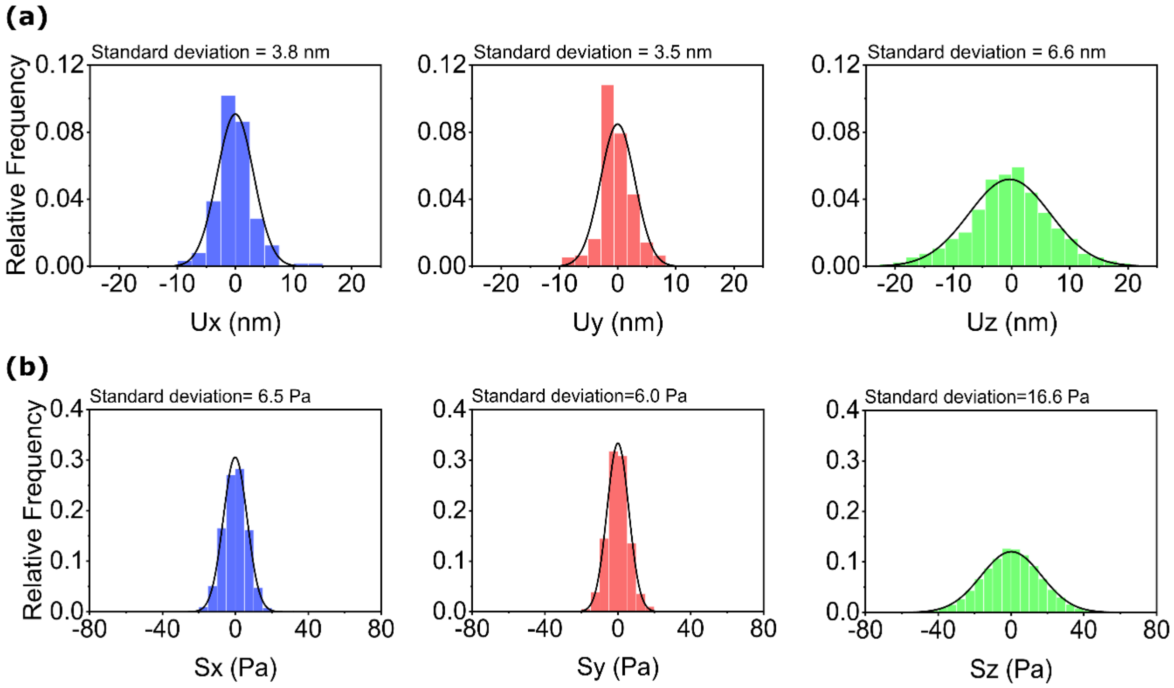

Supplementary Figure S12. (a) *Estimation of the displacement uncertainty. Plots show the lateral displacements uncertainty (blue and red histograms) as well as the axial displacement uncertainty (green histogram). The displacement uncertainty is calculated for the case of no cellular fluorescent background. The solid curves show a Gaussian fit.* ***(b)*** *Estimation of the corresponding uncertainty in the lateral (Sx, Sy) and axial (Sz) stresses using finite element simulations.*

It is worth noting that as Plotnikov, Sergey V., et al.[1] have demonstrated, displacement noises may generate huge errors in reconstructed stresses. Adding this effect to the Poisson's ratio error would even further decreases the accuracy of force reconstruction. However, using super-resolution modalities one can improve the process of ascertaining the position of beads and reduce the uncertainty regarding noise displacement. In our current study, coupling TIRF-SIM microscopy (to gain high resolution in lateral directions) and fast single-frame astigmatic imaging (to gain high axial resolution using a 100X objective) the standard deviation of the noises was reduced to ~4 and 7 nm in lateral and axial directions, respectively. These values are lower than those reported in[1], in which an epifluorescence microscope (with a 60X objective) was utilised that gave a noise error of 12 nm in lateral direction. Accordingly, the uncertainty in stress reconstruction is also smaller than that in[1]. Considering the magnitude of normal and shear tractions exerted by RBL and HeLa cell (**Fig. 3b** and **3f**, respectively), the errors stemmed from displacement noise are not significant in our study.

Supplementary Note 7- Selection of a representative value for Poisson's ratio

As mentioned in the main text, the Poisson's ratio of hydrogels is a time-dependent parameter and this time dependency rely on the poroelastic diffusion coefficient of the material. In poroelastic materials the time scale of mechanical events is related to the square of the involved length scale through $t \sim L^2/D_p$. In this note, based on the time and length scales involved during cellular force generation, we simply justify the use of asymptotic Poisson's ratio for the linear-elastic TFM analysis. The length scale for the strip is L_1=1mm (**Fig. S13a)**, and **Fig. 1h** illustrates that Poisson's ratio reaches to its asymptotic value within a time scale of t_1=300 s. On the other hand, for a TFM experiment, the length scale, which is the size of the loading region, could conservatively be estimated by the size of the cell (**Fig S13.b** and **c**). Now, we can write:

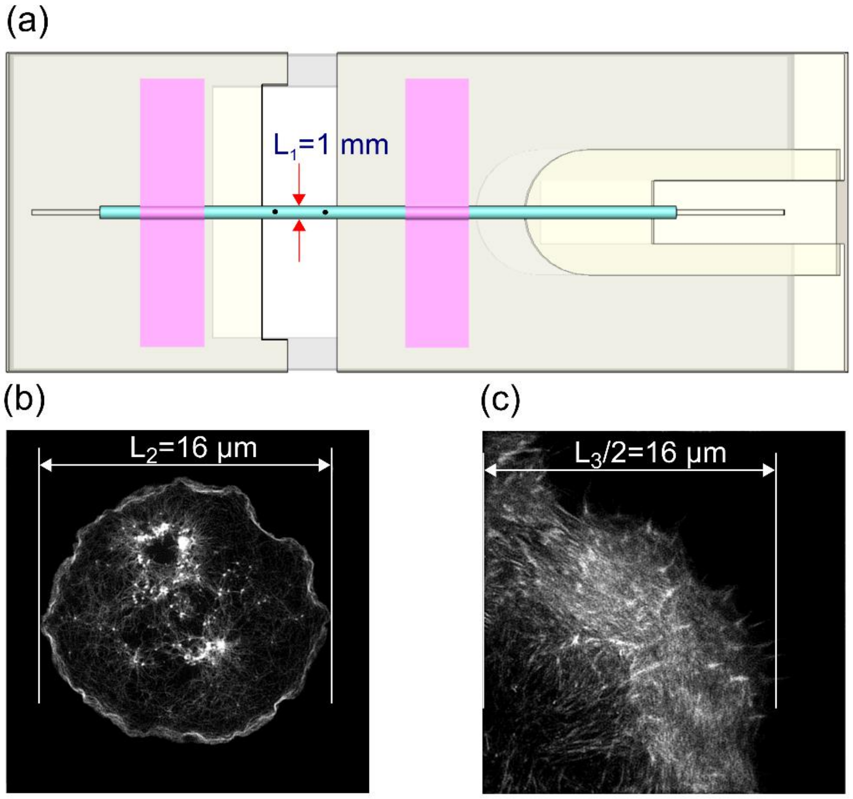

Supplementary Figure S13*. Length scales involving:* ***(a)*** *a Poisson's ratio quantification experiment* ***(b)*** *TFM experiment for an RBL cell (c) TFM experiment for a HeLa cell*

$$t \propto L^2/D_p \Rightarrow \frac{t_1}{L_1^2} = \frac{t_2}{L_2^2} = \frac{t_3}{L_3^2} \Rightarrow \frac{300}{1000^2} = \frac{t_2}{16^2} = \frac{t_3}{32^2} \Rightarrow \begin{cases} t_2 = 0.08\ s \\ t_3 = 0.30\ s \end{cases} \qquad \text{(S25)}$$

Eq. S25, indicates that for the RBL and HeLa cell, the Poisson's ratio reaches to its asymptotic value within 0.08 s and 0.3 s, respectively. In other words, the Poisson's ratio decreases within a fraction of a second, while normally the cellular force generation occurs during 1-10 s for the fastest cellular processes. This implies that asymptotic Poisson's ratio would provide the most relevant value to be used in a linear elastic TFM analysis. Nevertheless, a poroelastic model describes the mechanical behaviour of the hydrogel more precisely.

Supplementary References

1. Plotnikov, S. V., Sabass, B., Schwarz, U. S. & Waterman, C. M. High-Resolution Traction Force Microscopy. in 367–394 (2014). doi:10.1016/B978-0-12-420138-5.00020-3

2. Öchsner, A. *Classical Beam Theories of Structural Mechanics*. (Springer International Publishing, 2021). doi:10.1007/978-3-030-76035-9

3. Lurie, A. I. & Belyaev, A. *Theory of Elasticity*. (Springer Berlin Heidelberg, 2005). doi:10.1007/978-3-540-26455-2

4. Schwarz, U. S. & Soiné, J. R. D. Traction force microscopy on soft elastic substrates: A guide to recent computational advances. *Biochim. Biophys. Acta* **1853**, 3095–104 (2015).

5. Colin-York, H. *et al.* Spatiotemporally Super-Resolved Volumetric Traction Force Microscopy. *Nano Lett.* **19**, 4427–4434 (2019).

6. Bathe, K.-J. Finite Element Method. in *Wiley Encyclopedia of Computer Science and Engineering* (John Wiley & Sons, Inc., 2008). doi:10.1002/9780470050118.ecse159

7. Huang, Y. *et al.* Traction force microscopy with optimized regularization and automated Bayesian parameter selection for comparing cells. *Sci. Rep.* **9**, 539 (2019).

8. Sune-Aunon, A., Jorge-Penas, A., Van Oosterwyck, H. & Munoz-Barrutia, A. L1-regularized reconstruction for traction force microscopy. in *2016 IEEE 13th International Symposium on Biomedical Imaging (ISBI)* 140–144 (IEEE, 2016). doi:10.1109/ISBI.2016.7493230

9. Colin-York, H. *et al.* Super-Resolved Traction Force Microscopy (STFM). *Nano Lett.* (2016). doi:10.1021/acs.nanolett.6b00273

10. Burman, E., Hansbo, P. & Larson, M. G. Solving ill-posed control problems by stabilized finite element methods: an alternative to Tikhonov regularization. *Inverse Probl.* **34**, 035004 (2018).
